# Multi-Compartment Metabolic Remodeling in DRA-Deficient Mice Overlaps with Ulcerative Colitis Metabolic Signatures

**DOI:** 10.3390/metabo16090690

**Published:** 2026-09-18

**Authors:** Jayson M. Antonio, Shubha Priyamvada, Arivarasu N. Anbazhagan, Harris J. B., Nathan Calzadilla, Rengul C. Atalay, Reena Sethi, Seema Saksena, Ravinder K. Gill, Gokhan M. Mutlu, Waddah A. Alrefai, Ece A. Mutlu, Anoop Kumar, Pradeep K. Dudeja

**Affiliations:** 1 Division of Gastroenterology and Hepatology, Department of Medicine, University of Illinois Chicago, Chicago, IL 60612, USA; jma429@uic.edu (J.M.A.); shubha@uic.edu (S.P.); arivu29@uic.edu (A.N.A.); jharri87@uic.edu (H.J.B.); walrefai@uic.edu (W.A.A.); emutlu@uic.edu (E.A.M.); anoop@uic.edu (A.K.); 2 Section of Plastic and Reconstructive Surgery, Department of Surgery, The University of Chicago, Chicago, IL 60637, USA; nathan.calzadilla@uchicagomedicine.org; 3 Section of Pulmonary and Critical Care Medicine, Department of Medicine, The University of Chicago, Chicago, IL 60637, USA; rengul@uchicago.edu (R.C.A.); gokhan.mutlu@uchicagomedicine.org (G.M.M.); 4 Department of Biology, Virginia Commonwealth University, Richmond, VA 23284, USA; reenasethi1122@gmail.com; 5 Jesse Brown VA Medical Center, Chicago, IL 60612, USA; saksena@uic.edu (S.S.); rgill@uic.edu (R.K.G.)

**Keywords:** epithelial ion transport, chloride-bicarbonate exchange, inflammatory bowel disease, LC–MS metabolomics, RNA sequencing, bile acid profiling, host-microbiota co-metabolism

## Abstract

**Highlights:**

**What are the main findings?**

• Lower SLC26A3 expression across six ulcerative colitis (UC) cohorts was associated with broad suppression of inferred metabolic pathway activity.
• DRA-deficient mice showed broad metabolic changes across colonic mucosa, feces, and serum, involving amino acid, redox, polyamine, and bile acid metabolism

**What are the implications of the main findings?**

• DRA loss may disrupt epithelial–luminal metabolic homeostasis beyond chloride–bicarbonate exchange.
• Metabolic pathway disturbances accompanying DRA loss may reflect a dysregulated homeostatic epithelial-luminal environment relevant to intestinal inflammation.

**Abstract:**

**Background/Objectives**: Down-regulated in adenoma (DRA/SLC26A3), an IBD (Inflammatory Bowel Diseases) susceptibility gene and major colonic Cl^−^/HCO_3_^−^ exchanger, is reduced in ulcerative colitis (UC). Whether reduced DRA is linked to broader metabolic remodeling during intestinal inflammation remains unclear. We therefore examined DRA-associated metabolic pathways in human UC datasets and metabolic changes across mucosal, luminal, and systemic compartments in DRA-deficient mice. **Methods**: Six publicly available human colonic transcriptomic cohorts were analyzed to infer KEGG metabolic pathway activity, compare UC with healthy mucosa, and assess associations with DRA expression using random-effects meta-analysis. DRA knockout mice were evaluated by colonic mucosal RNA sequencing, untargeted LC–MS metabolomics of colonic mucosa, feces, and serum, targeted bile acid profiling, MetOrigin analysis, and fecal 16S rRNA sequencing. **Results**: In the human cohorts, DRA expression was consistently lower in UC. Of the 75 KEGG metabolic pathways evaluated, 48 were altered in UC relative to healthy mucosa, and 45 of these were also associated with DRA expression levels among UC samples. In DRA knockout mice, colonic RNA sequencing identified broad metabolic transcriptional remodeling across 39 KEGG pathways, with metabolic themes that overlapped with the human UC findings. Multi-compartment metabolomics further revealed alterations involving amino acid, nitrogen, redox/cofactor, polyamine, and bile acid metabolism, together with inferred host-microbiota co-metabolic signatures. **Conclusions**: DRA deficiency was accompanied by broad metabolic remodeling across mucosal, luminal, and systemic compartments. These changes overlapped with DRA-associated signatures in human UC, supporting a role for DRA in epithelial–luminal metabolic homeostasis beyond ion transport.

## 1. Introduction

Maintaining electrolyte absorption, secretion, and luminal pH in the gastrointestinal tract requires the coordinated activity of epithelial ion transporters, including chloride-bicarbonate (Cl^−^/HCO_3_^−^) exchangers [1,2,3]. Genetic defects in these transporters, as well as their dysregulation during infectious or inflammatory diarrhea, can impair epithelial ion flux and contribute to intestinal fluid loss [1,4,5,6,7]. Among these transporters, down-regulated in adenoma (DRA; SLC26A3) is the major apical Cl^−^/HCO_3_^−^ exchanger in the colon and is essential for intestinal fluid and electrolyte homeostasis [1,3,6]. Loss-of-function mutations in SLC26A3 cause congenital chloride diarrhea (CLD), characterized by lifelong chloride-rich diarrhea and metabolic alkalosis [7]. Similarly, DRA knockout (KO) mice develop watery, high-chloride diarrhea and provide a useful model for studying the broader consequences of DRA deficiency [6].

Beyond its essential role in intestinal ion transport, genetic and clinical evidence implicates DRA in inflammatory bowel disease susceptibility [8,9,10,11]. A genome-wide association study in a Japanese population identified a susceptibility signal within the SLC26A3 locus, represented by rs2108225, for ulcerative colitis (UC) [8]. A subsequent trans-ancestry analysis further supported this locus, showing association of rs2108225 in the East Asian cohort and a strong association with IBD in the European cohort [11]. A study in Chinese patients also found that rs2108225 may increase UC risk and affect SLC26A3 mRNA and protein expression in colonic tissues [9]. In addition, patients with CLD caused by SLC26A3 mutations exhibit an increased incidence of inflammatory bowel disease [10]. Together, these findings support SLC26A3/DRA as an IBD susceptibility locus and suggest that reduced DRA expression or function may contribute to disease risk.

Several consequences of DRA deficiency may provide a biological basis for this association. Loss of DRA compromises epithelial junctional organization and barrier integrity [12], reduces colonic bicarbonate secretion and prevents formation of a firm mucus layer [13], alters the colonic microbiota [12,14], and disrupts mucosal immune homeostasis through epithelial–immune cell crosstalk [15]. DRA-deficient mice also show increased susceptibility to experimental mucosal injury and colitis [12,13,15]. Together, these effects suggest that reduced DRA function creates a transport-defective, structurally weakened, immunologically altered, and dysbiotic intestinal environment that may favor the development or persistence of inflammation.

Despite this evidence, whether the epithelial, immune, and microbial abnormalities associated with DRA deficiency are accompanied by broader metabolic remodeling remains poorly defined. It is unclear whether reduced DRA expression is associated with altered metabolic pathway activity in human UC and whether genetic DRA deficiency produces coordinated transcriptional and metabolomic changes in the colonic mucosa. It is also unknown whether these alterations extend to the luminal and systemic compartments or reflect contributions from host metabolism, microbial metabolism, or host-microbiota co-metabolism.

In the present study, we investigated the relationship between DRA/SLC26A3 and metabolic remodeling in human UC and DRA KO mice. We first performed pathway-level analyses across six independent, publicly available human colonic transcriptomic cohorts to identify metabolic pathways altered in UC and determine whether these pathways varied with DRA expression levels among UC samples. We then combined metabolism-focused colonic transcriptomic analysis in DRA KO mice with untargeted metabolomics of colonic mucosa, feces, and serum, targeted bile acid profiling of serum and feces, and metabolite-origin analysis. This design allowed us to ask whether the metabolic pathway patterns associated with reduced DRA expression in UC converge with metabolic changes observed after genetic DRA loss in mice. Our findings reveal broad metabolic pathway alteration in human UC, strong association between UC-altered pathways and DRA expression levels, coordinated metabolic transcriptional and metabolomic remodeling in DRA-deficient colonic mucosa, prominent amino acid and polyamine-related alterations in mucosal and fecal compartments, compartment-specific bile acid changes, and inferred host-microbiota co-metabolic signatures. Together, these results support a broader role for DRA/SLC26A3 in linking epithelial ion transport with mucosal metabolic homeostasis, luminal metabolism, bile acid regulation, and gut inflammation-associated remodeling.

## 2. Materials and Methods

The methods summarized below describe the principal experimental and analytical procedures. Detailed protocols, cohort-curation procedures, analysis parameters, sensitivity analyses, and derived summary tables are available in the accompanying Zenodo repository (https://doi.org/10.5281/zenodo.21704235).

### 2.1. Human UC Transcriptomic Pathway Analysis

Six publicly available human colonic transcriptomic cohorts were analyzed in silico: GSE11223 [16], GSE38713 [17], GSE206171 [18], GSE206285 [19], GSE53306 [20], and GSE59071 [21]. Expression data and phenotype metadata were processed independently within each cohort, and SLC26A3 expression was used as the transcriptomic measure of DRA expression.

Since these cohorts differed in transcriptomic platform, preprocessing method, expression scale, tissue sampling, disease characteristics, and sample composition, raw expression values and raw pathway scores were not pooled across datasets. Instead, pathway scoring and statistical modeling were performed within each cohort before cross-cohort integration. Cohort-specific standardized effect estimates, and standard errors were then combined using random-effects meta-analysis with restricted maximum likelihood estimation. For GSE206171, paired sigmoid biopsies from the same participant were averaged to participant-level profiles before pathway scoring and statistical modeling. For GSE11223, terminal ileal samples were excluded; colonic region and patient structure were accounted for in the UC-versus-healthy/control model; and the locked descending-colon subset was used for the final DRA-focused expression and within-UC pathway-association analyses. Additional cohort-curation and model-specification details are provided in the accompanying detailed methods repository.

Seventy-five human KEGG metabolic pathways were defined from subpathways linked to the hsa01100 “Metabolic pathways” reference map [22]. Single-sample gene set enrichment analysis was performed separately within each cohort using GSVA version 2.4.9 to infer transcriptional pathway activity [23]. UC-versus-healthy/control effects were estimated within each cohort and pooled using random-effects meta-analysis with restricted maximum likelihood estimation implemented using metafor version 5.0.1 [24]. DRA expression was evaluated across the same cohorts. Within UC samples, associations between DRA expression and inferred pathway activity were estimated separately by cohort and pooled using random-effects meta-analysis [24]. DRA-associated pathway effects were compared with independently derived UC-versus-healthy/control effects to evaluate directional concordance. These analyses were interpreted as observational associations rather than causal tests.

### 2.2. Animal Studies and Sample Collection

All animal studies were approved by the Institutional Animal Care and Use Committees of the Jesse Brown Veterans Affairs Medical Center and the University of Illinois Chicago. Wild-type and Slc26a3-deficient DRA KO mice, aged 8–10 weeks, were maintained under specific pathogen-free conditions, with sex composition specified by assay cohort below. All animals, including both WT and DRA KO mice, received an oral electrolyte solution to reduce dehydration risk in DRA KO mice and to avoid comparing dehydrated DRA KO animals with normally hydrated WT controls [6,12]. Thus, electrolyte exposure was applied to both genotypes and was not a genotype-specific treatment. Colonic mucosal RNA sequencing was performed using WT and DRA KO mice with n = 3 biological replicates per genotype; this RNA-seq cohort consisted of male mice only. Untargeted LC-MS metabolomics of colonic mucosa, feces, and serum was performed using an independent WT and DRA KO cohort with n = 5 biological replicates per genotype, including n = 3 male and n = 2 female mice per genotype. Targeted bile acid profiling of serum and feces was performed using a separate WT and DRA KO mouse cohort with n = 5 biological replicates per genotype; this bile acid cohort consisted of male mice only. Fecal 16S rRNA sequencing was performed using a separate WT and DRA KO mouse cohort with n = 5 biological replicates per genotype, including n = 3 male and n = 2 female mice per genotype.

Mice were generated from heterozygous crosses to control for parentage, in utero environment, and early-life environmental exposure. After genotyping, WT and DRA KO mice were housed separately by sex and genotype for at least 4 weeks before sacrifice, allowing genotype-associated physiological and microbiome features to develop under standardized housing conditions. Multiple independent sets of WT and DRA KO mice were used for distinct experimental assays to reduce the influence of cage-specific effects on any single experimental outcome.

### 2.3. Mouse RNA-Seq and Metabolism-Focused Transcriptomics

RNA was isolated from colonic mucosa, and strand-specific libraries were sequenced on an Illumina HiSeq 4000 platform (Illumina, Inc., San Diego, CA, USA). Reads were aligned to the mouse GRCm39 reference genome using STAR version 2.7 [25], and differential expression between DRA KO and wild-type mice was assessed using DESeq2 version 1.50.2 [26]. Genes with FDR < 0.05 were considered differentially expressed.

Testable genes were mapped to KEGG metabolic pathways [22], and gene set enrichment analysis was performed using the DESeq2 Wald statistic-ranked gene list [26]. Variance-stabilized expression values were used for metabolism-focused principal component analysis, pathway scoring, gene-loading analysis, and pathway-level summaries.

### 2.4. Untargeted LC–MS Metabolomics and Pathway Analysis

Untargeted metabolomics of colonic mucosa, feces, and serum was performed by the Southeast Center for Integrated Metabolomics (SECIM, University of Florida, Gainesville, FL, USA) using positive- and negative-ion LC–MS. Raw data were processed using MZmine for feature detection, alignment, and preliminary annotation [27]. Feature-intensity data were normalized using probabilistic quotient normalization [28], followed by log transformation and Pareto scaling. Multivariate and univariate analyses were performed using MetaboAnalyst 6.0 and MetaboAnalystR version 4.3.0 [29,30]. Differential metabolite features were defined using FDR < 0.05 and a DRA-knockout-to-wild-type fold change ≥ 1.30 or ≤0.769. Variable importance in projection scores was considered together with statistical significance, fold change, annotation confidence, and pathway context. Since PLS-DA is a supervised method and can overfit in small high-dimensional metabolomic datasets, PLS-DA models were used only for feature prioritization and visualization rather than as the primary basis for statistical inference. Model performance was evaluated using 5-fold cross-validation to estimate R^2^ and Q^2^ values, and overfitting was assessed using permutation testing with 1000 permutations. The PLS-DA component with the highest Q^2^ value was used for VIP-ranked feature prioritization. Metabolite features were considered significantly altered only when they met the univariate criteria of FDR < 0.05 and KO-to-WT fold change ≥ 1.30 or ≤0.769; VIP score alone was not used to define differential abundance.

Pathway analysis was performed against the Mus musculus KEGG pathway library using complementary metabolite-annotation layers. Metabolite identification confidence was interpreted according to Metabolomics Standards Initiative criteria [31]. Pathways with FDR < 0.05 were considered significant, whereas less confident annotation layers were treated as exploratory. Detailed metabolomics quality-control procedures, feature filtering criteria, preprocessing parameters, and MSI annotation workflow are provided in the accompanying detailed methods repository and processed LC-MS repository tables.

### 2.5. Transcriptome-Metabolome Pathway-Level Convergence

Because the colonic transcriptomic and metabolomic datasets were generated from independent mouse cohorts, integration was performed at the pathway level rather than through sample-wise gene–metabolite correlations. Metabolism-associated genes differentially expressed at FDR < 0.05 and high-confidence metabolites significantly altered at FDR < 0.05 were mapped to KEGG pathways [22,27]. Pathways supported by both transcriptomic and metabolomic evidence were interpreted as showing pathway-level convergence.

### 2.6. Targeted Bile Acid Profiling, MetOrigin Analysis, and 16S rRNA Profiling

Targeted bile acid profiling was performed in serum and fecal samples by LC–MS/MS at the McGuire VAMC Research Institute Mass Spectrometry Facility (Richmond, VA, USA) [32]. Bile acids were evaluated individually and according to origin, conjugation state, sulfation status, and pathway-related summary measures.

Differential metabolite features were analyzed using MetOrigin 2.0 to infer possible host-associated, microbiota-associated, host–microbiota co-metabolic, dietary, drug-associated, or environmental origins [33]. Fecal microbial communities were profiled by 16S rRNA gene sequencing of the V3–V4 region using the ZymoBIOMICS Targeted Sequencing Service (Zymo Research Corporation, Irvine, CA, USA). Amplicon sequence variants were inferred using DADA2 [34], and taxonomy was assigned using Uclust in QIIME v1.9.1 [35] against the Zymo Research Database. Genus-level relative abundances were used for descriptive comparisons with MetOrigin-inferred co-metabolic signatures.

### 2.7. Statistical Analysis

Analyses were performed using R version 4.5.2 unless otherwise specified. Human pathway scoring used GSVA version 2.4.9 [23], mixed-effects models used nlme version 3.1-168 [36], and random-effects meta-analysis used metafor version 5.0.1 with restricted maximum likelihood estimation [24]. Mouse RNA-seq differential expression was performed using DESeq2 version 1.50.2 [26]. Genes with Benjamini–Hochberg FDR < 0.05 were considered differentially expressed. 

KEGG pathway definitions and gene annotations were generated using KEGGREST version 1.50.0, AnnotationDbi version 1.72.0, org.Hs.eg.db version 3.22.0, and org.Mm.eg.db version 3.22.0 where applicable [22]. GSVA/ssGSEA-derived KEGG pathway scores were interpreted as inferred transcriptional pathway activity scores. These scores do not directly measure metabolic flux, metabolite abundance, or biochemical enzyme activity. Therefore, all human transcriptomic pathway analyses were interpreted as transcriptional pathway associations. 

Data processing and visualization used dplyr version 1.2.1, tidyr version 1.3.2, readr version 2.2.0, purr version 1.2.2, tibble version 3.3.0, ggplot2 version 4.0.2, ggrepel version 0.9.6, and ComplexHeatmap version 2.26.1 where applicable [37]. Untargeted metabolomics analyses were performed using MetaboAnalyst 6.0 and MetaboAnalystR where applicable [29]. Source-attribution and co-metabolism analyses were performed using MetOrigin 2.0 [33]. Unless otherwise specified, multiple-testing correction was performed using the Benjamini-Hochberg method [38], and FDR < 0.05 was considered statistically significant.

### 2.8. Use of Generative AI and AI-Assisted Technologies

ChatGPT 5.2 was used solely to assist with troubleshooting R code and was not used to generate scientific interpretations or conclusions. All analytical decisions and code execution were performed by authors. Statistical outputs and figures were independently reviewed and verified by the authors, and all scientific interpretations and conclusions were developed by the authors.

## 3. Results

### 3.1. Cross-Cohort Analysis Identifies Metabolic Pathway Alterations Linked to Reduced DRA Expression in UC

**Figure 1:**
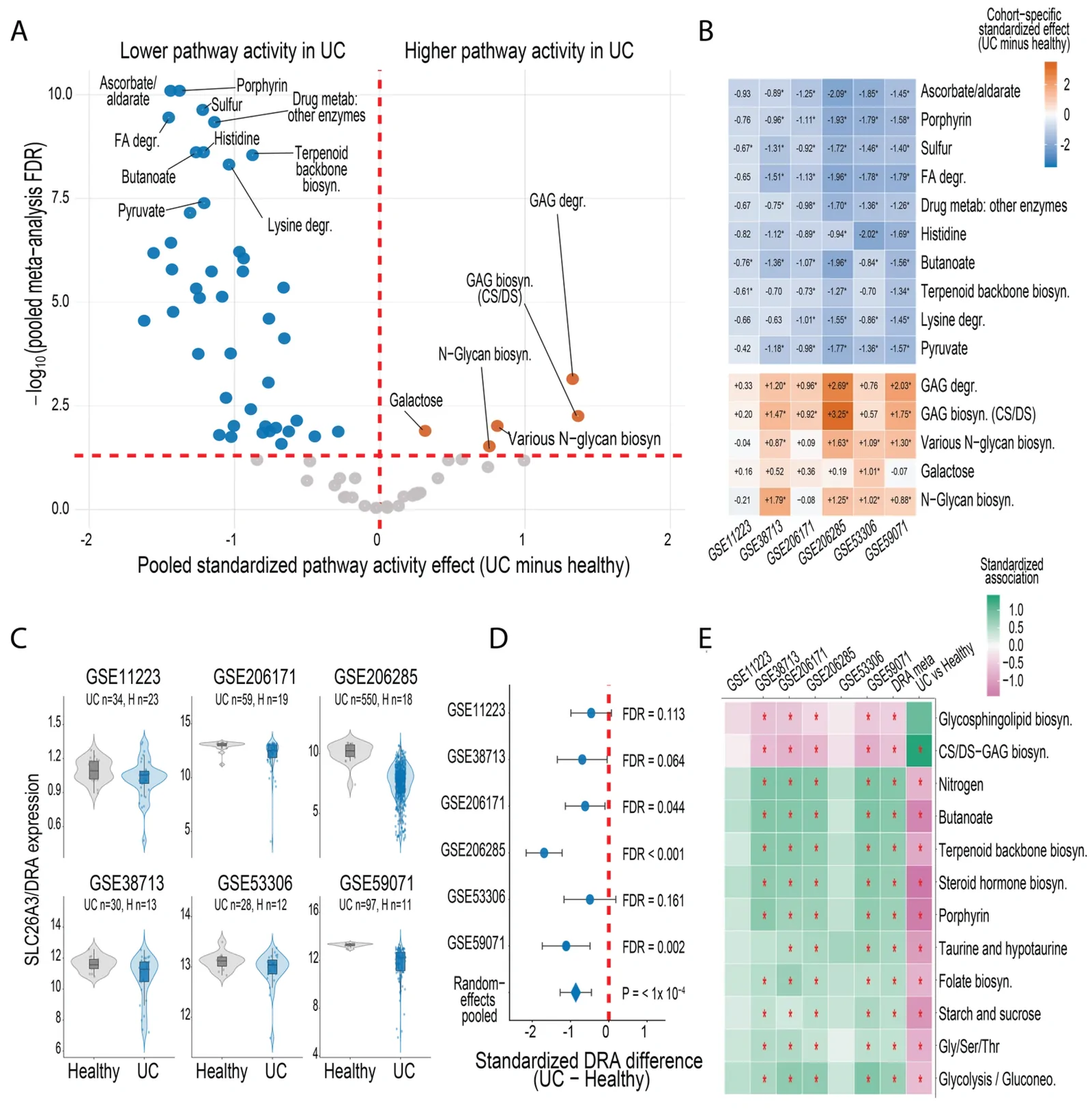
**Cross-cohort analysis identifies UC-associated metabolic pathway alterations linked to reduced DRA expression.** Six independent human colonic transcriptomic cohorts were analyzed: GSE11223, GSE38713, GSE206171, GSE206285, GSE53306, and GSE59071. Pathway activity was inferred using KEGG metabolic pathway scores, and cohort-level effects were pooled using random-effects meta-analysis. FDR was calculated using Benjamini-Hochberg correction. (**A**) Volcano plot showing pooled standardized UC versus healthy/control effects across 75 KEGG metabolic pathways. The x-axis shows the pooled standardized pathway activity effect, and the y-axis shows −log_10_ FDR from the pooled meta-analysis. Blue points indicate pathways significantly lower in UC, orange points indicate pathways significantly higher in UC, and gray points indicate non-significant pathways. (**B**) Heatmap of 15 highlighted pathways, including representative pathways reduced in UC and all pathways increased in UC. Values indicate cohort-specific standardized UC minus healthy/control effects. Asterisks indicate cohort-level FDR < 0.05. (**C**) Violin plots showing SLC26A3/DRA expression in healthy/control and UC samples across the six cohorts. Boxplots and individual samples are overlaid; sample sizes are shown above each panel. (**D**) Forest plot of cohort-specific and random-effects pooled standardized DRA expression differences between UC and healthy/control mucosa. Negative values indicate lower DRA expression in UC. The diamond represents the pooled estimate. Cohort-level FDR values are shown to the right, and the pooled meta-analysis P value is shown for the overall estimate. (**E**) Heatmap of representative KEGG pathways associated with DRA expression levels within UC samples. The first six columns show cohort-specific standardized associations between pathway activity and DRA expression among UC samples only. The DRA meta column shows the random-effects pooled association between DRA expression levels and pathway activity across the six UC cohorts. The UC vs. Healthy column shows the independently derived random-effects pooled UC-versus-healthy/control pathway effect for comparison. Green indicates positive effects or associations, and pink indicates negative effects or associations. Asterisks indicate FDR < 0.05. **Abbreviations:** UC, ulcerative colitis; H, healthy/ control DRA, down-regulated in adenoma; SLC26A3, solute carrier family 26 member 3; KEGG, Kyoto Encyclopedia of Genes and Genomes; FDR, false discovery rate; GAG, glycosaminoglycan; CS/DS, chondroitin sulfate/dermatan sulfate; FA, fatty acid; Gly/Ser/Thr, glycine/serine/threonine metabolism; Gluconeo., gluconeogenesis; biosyn., biosynthesis; degr., degradation; Drug metab., drug metabolism; DRA meta, random-effects pooled DRA-pathway association across UC samples.

To systematically characterize metabolic remodeling in ulcerative colitis (UC), we performed a targeted cross-cohort analysis of KEGG metabolic pathways across six independent human colonic transcriptomic cohorts: GSE11223, GSE38713, GSE206171, GSE206285, GSE53306, and GSE59071. We focused specifically on metabolism and evaluated all 75 KEGG metabolic pathways to obtain a comprehensive and consistent assessment of inferred transcriptional activity of metabolic pathways across cohorts. Among the 75 KEGG metabolic pathways evaluated, 48 showed significant pooled differences in inferred transcriptional pathway activity between UC and healthy/control mucosa after FDR correction. The pathway changes were predominantly decreased in UC, with 43 pathways showing lower inferred transcriptional activity and only five showing higher activity (Figure 1A; Supplementary Figure S1A).

The pathways reduced in UC were not limited to a single metabolic class, but instead suggested a broad reduction in colonic metabolic programs. The strongest changes involved redox and cofactor metabolism, lipid utilization and xenobiotic handling, selected amino acid pathways, and central energy metabolism, with representative alterations in ascorbate/aldarate, porphyrin, sulfur, fatty acid degradation, drug metabolism by other enzymes, butanoate, and pyruvate metabolism. In contrast, the five pathways increased in UC were enriched for glycan biosynthesis and remodeling, together with galactose metabolism (Figure 1A,B; Supplementary Figure S1A).

Given the central role of DRA in colonic epithelial ion transport and IBD, we next examined whether SLC26A3 expression was consistently altered across the same UC cohorts. DRA expression showed a uniform reduction in UC across all six datasets (Figure 1C). This reduction reached cohort-level FDR significance in GSE206171, GSE206285, and GSE59071, while the remaining cohorts showed concordant lower expression. Importantly, random-effects meta-analysis confirmed a significant overall reduction in DRA expression in UC (pooled standardized effect = −0.862, 95% CI: −1.271 to −0.454, *p* < 0.001; Figure 1D). Sensitivity analyses confirmed that the overall reduction in DRA expression was robust. The pooled effect remained negative when each cohort was removed one at a time and when group sizes were balanced by repeated resampling. GSE206285 contributed most strongly to the magnitude of the pooled estimate, but removing individual cohorts did not change the overall direction of the result (Supplementary Figure S1B–D).

We then focused only on UC samples and asked whether the disease-altered pathways varied according to DRA expression. Of the 48 pathways altered in the UC versus healthy/control comparison, 45 also varied with DRA expression among UC samples. Given the bulk mucosal nature of these datasets, these associations should be interpreted as features of a DRA-associated UC mucosal profile rather than as DRA-specific effects. All 43 pathways with lower activity in UC showed higher activity in UC samples with higher DRA expression, suggesting that higher DRA expression is linked to higher inferred transcriptional activity of metabolic programs that are generally reduced in UC. This pattern was also evident among representative DRA-associated pathways, where higher DRA expression aligned with increased activity of interconnected metabolic processes involved in carbon utilization, nitrogen and amino acid handling, cofactor/redox balance, and short-chain fatty acid-related metabolism (Figure 1E). In contrast, selected glycan-related pathways, including chondroitin sulfate/dermatan sulfate glycosaminoglycan (CS/DS-GAG) biosynthesis and glycosphingolipid biosynthesis, showed inverse associations with DRA expression. Across the full within-UC DRA analysis, 57 pathways varied with DRA expression, including 45 pathways shared with the UC-versus-healthy metabolic signature (Supplementary Figure S1E). Overall, these findings indicate that lower DRA expression within UC samples is associated with broader inferred metabolic pathway suppression and selected glycan-related remodeling.

### 3.2. DRA Deficiency Reveals Coordinated Remodeling of the Colonic Metabolic Transcriptome

**Figure 2:**
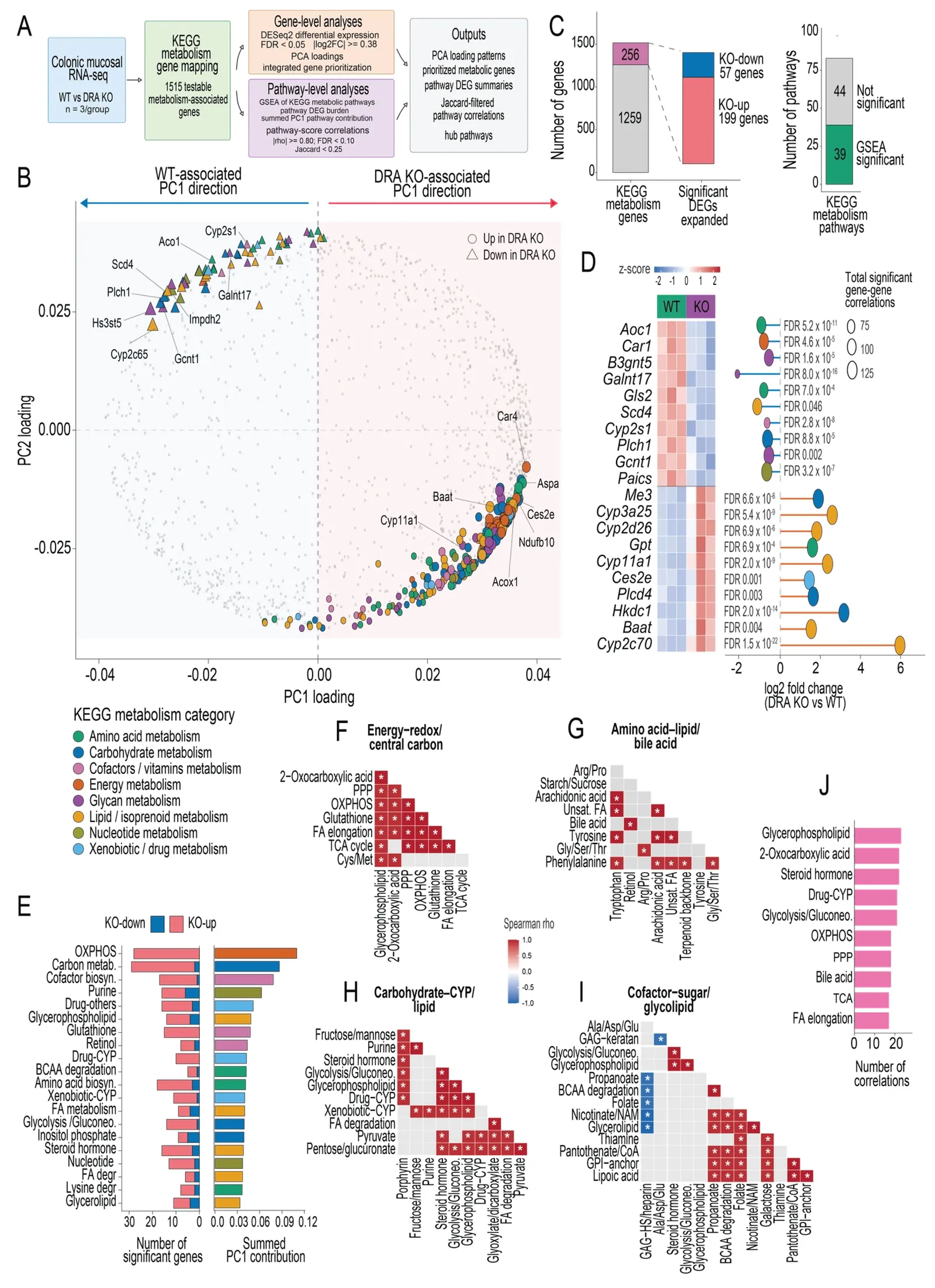
**DRA deficiency reveals coordinated remodeling of the colonic metabolic transcriptome.** Colonic RNA-seq data from wild-type (WT) and DRA knockout (KO) mice were analyzed using n = 3 biological replicates per genotype and a metabolism-focused framework restricted to genes assigned to KEGG metabolic pathways. (**A**) Workflow for the metabolism-focused colonic RNA-seq analysis. Colonic mucosal RNA-seq profiles from WT and DRA KO mice were mapped to KEGG metabolism-associated genes and analyzed using gene-level and pathway-level approaches. Gene-level analyses included DESeq2 differential-expression testing, PCA loading analysis, and integrated gene prioritization. Pathway-level analyses included GSEA of KEGG metabolic pathways, pathway DEG burden, summed PC1 pathway contribution, pathway-score correlations, Jaccard overlap filtering, and hub pathway identification. (**B**) PCA loading plot of KEGG metabolism-associated genes showing WT-associated and DRA KO-associated PC1 directions. Each point represents one gene. Gray points indicate non-significant genes, whereas significant metabolic DEGs are shown according to broad KEGG metabolic category. Circles indicate genes increased in DRA KO, and triangles indicate genes decreased in DRA KO relative to WT. (**C**) Summary of testable KEGG metabolism-associated genes, significant metabolic DEGs, and significant KEGG metabolism pathways identified by GSEA. KO-up genes indicate genes increased in DRA KO colon, whereas KO-down genes indicate genes decreased in DRA KO colon. (**D**) Prioritized metabolic differentially expressed genes selected based on differential-expression significance, absolute log2 fold change, absolute PC1 loading, and gene-gene correlation connectivity. The heatmap shows gene-wise z-scored expression across WT and DRA KO samples, and the adjacent plot shows log_2_ fold change in DRA KO versus WT. Point size indicates the number of significant gene-gene correlations with other metabolic DEGs. (**E**) Pathway-level summary integrating DEG burden and contribution to the genotype-associated PC1 direction. Bars indicate the number of KO-up and KO-down metabolic DEGs per pathway, together with the summed PC1 contribution of pathway genes. (**F**–**I**) Representative groups of interrelated KEGG metabolism pathways identified from pathway-score correlation analysis. Pathway scores were correlated across samples, and retained correlations met thresholds for strong association and limited gene-set overlap. Asterisks indicate FDR < 0.05. (**J**) Top pathway-correlation hubs ranked by the number of retained pathway correlations. Highly connected pathways include glycerophospholipid metabolism, 2-oxocarboxylic acid metabolism, steroid hormone biosynthesis, drug metabolism by cytochrome P450, glycolysis/gluconeogenesis, oxidative phosphorylation, pentose phosphate pathway, primary bile acid biosynthesis, tricarboxylic acid cycle, and fatty acid elongation. **Abbreviations:** WT, wild-type; KO, knockout; DRA, down-regulated in adenoma; KEGG, Kyoto Encyclopedia of Genes and Genomes; DEG, differentially expressed gene; GSEA, gene set enrichment analysis; PCA, principal component analysis; PC1, principal component 1; PC2, principal component 2; FDR, false discovery rate; KO-up, increased in DRA KO versus WT; KO-down, decreased in DRA KO versus WT; OXPHOS, oxidative phosphorylation; PPP, pentose phosphate pathway; TCA cycle, tricarboxylic acid cycle; FA, fatty acid; Unsat. FA, unsaturated fatty acid; CYP, cytochrome P450; Drug-CYP, drug metabolism by cytochrome P450; Xenobiotic-CYP, xenobiotic metabolism by cytochrome P450; BCAA, branched-chain amino acid; GPI, glycosylphosphatidylinositol; GAG, glycosaminoglycan; HS, heparan sulfate; Gly/Ser/Thr, glycine/serine/threonine metabolism; Arg/Pro, arginine/proline metabolism; Cys/Met, cysteine/methionine metabolism; Ala/Asp/Glu, alanine/aspartate/glutamate metabolism; NAM, nicotinamide; CoA, coenzyme A; biosyn., biosynthesis; degr., degradation; metab., metabolism; Gluconeo., gluconeogenesis.

In light of the observations that DRA expression was linked to metabolic pathway activity in human UC cohorts, we next explored whether loss of DRA was associated with metabolic transcriptional remodeling in colonic mucosa. We performed a metabolism-focused analysis of colonic RNA-seq data from WT and DRA KO mice using n = 3 biological replicates per genotype, restricting the analysis to genes assigned to KEGG metabolism pathways (Figure 2A). Using this metabolism-focused gene set, we examined whether metabolic genes exhibited directional organization by genotype. The loading analysis showed opposing WT-associated and DRA KO-associated directions, suggesting that metabolism-related genes contributed to the separation between WT and DRA KO colonic mucosa rather than being randomly distributed (Figure 2B).

Consistent with this organized pattern, 256 of 1515 testable KEGG metabolism-associated genes were significantly differentially expressed between DRA KO and WT colonic mucosa, including 199 genes increased in DRA KO and 57 genes higher in WT or reduced in DRA KO (Figure 2C). At the pathway level, GSEA identified 39 significant KEGG metabolism pathways, indicating broad metabolic transcriptional remodeling in DRA KO colon. Because this RNA-seq analysis was performed with n = 3 biological replicates per genotype, these findings were interpreted as genotype-associated pathway-level patterns rather than definitive estimates of individual gene effects. Larger independent cohorts will be needed to refine the individual gene-level conclusions and validate the broader pathway-level patterns.

We next sought to identify metabolic DEGs most strongly involved in the DRA KO-associated transcriptional shift. To do this, we prioritized genes based on differential-expression strength, contribution to the genotype-associated direction, and the number of significant correlations with other metabolic DEGs (Figure 2D). This highlighted genes with lower expression in DRA KO colon, such as *Aoc1*, *Car1*, *B3gnt5*, *Galnt17*, *Gls2*, and *Scd4*, together with genes with higher expression in DRA KO colon, such as *Me3*, *Cyp3a25*, *Cyp2d26*, *Gpt*, *Cyp11a1*, and *Ces2e*. Several of these genes were also highly connected with other metabolic DEGs, suggesting that they represent prioritized components of the DRA deficiency-associated metabolic transcriptional pattern.

We then asked whether these gene-level changes converged on broader metabolic pathways. For each pathway, we summarized the number of DRA KO-high and DRA KO-low genes together with the summed contribution of pathway genes to the genotype-associated direction (Figure 2E). This pathway-level view highlighted metabolic themes that overlapped with the human DRA-associated signature (Figure 1), including energy and carbon metabolism, lipid remodeling, amino acid and nucleotide metabolism, redox/cofactor pathways, and xenobiotic/drug metabolism. Since pathway-level summaries can be influenced by shared pathway gene membership, we next examined whether pathway expression scores were coordinated across samples. After filtering for strong correlations with limited gene-set overlap, several metabolic pathways showed coordinated pathway-score relationships across the metabolism-focused network (Supplementary Figure S2). From this filtered correlation network, we selected representative groups of interrelated pathways for visualization, including energy-redox/central carbon metabolism, amino acid-lipid/bile acid metabolism, carbohydrate-CYP/lipid metabolism, and cofactor-sugar/glycolipid metabolism (Figure 2F–I). Within the same filtered network, pathway-hub analysis summarized the number of retained correlations per pathway, identifying glycerophospholipid metabolism, 2-oxocarboxylic acid metabolism, steroid hormone biosynthesis, Drug-CYP, glycolysis/gluconeogenesis, OXPHOS, PPP, primary bile acid biosynthesis, TCA cycle, and FA elongation among the most connected pathways (Figure 2J). Together, these findings extend the human UC analysis by showing that DRA deficiency in mouse colonic mucosa is associated with coordinated metabolic transcriptional organization centered on lipid remodeling, central carbon metabolism, energy-redox pathways, bile acid metabolism, and xenobiotic/steroid-associated pathways.

### 3.3. DRA Deficiency Is Accompanied by Coordinated Remodeling of the Colonic Mucosal Metabolome

**Figure 3:**
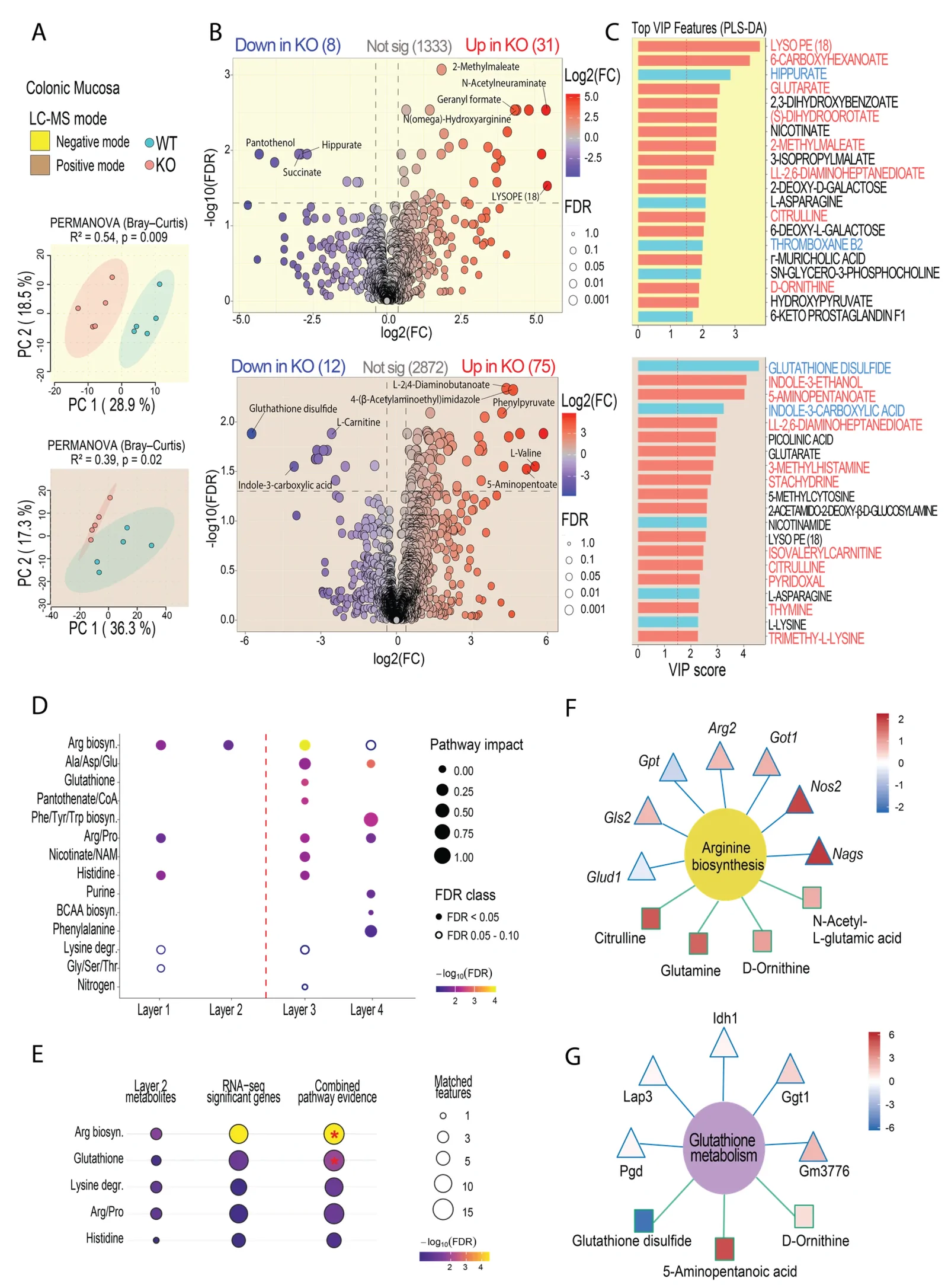
**DRA deficiency is accompanied by coordinated remodeling of the colonic mucosal metabolome.** Untargeted LC–MS metabolomics was performed on colonic mucosal samples from separate WT and DRA KO mouse cohort with n = 5 biological replicates per genotype in negative- and positive-ion modes. (**A**) Principal component analysis of colonic mucosal metabolite profiles acquired in negative-ion mode and positive-ion mode. Each point represents one biological replicate. WT and DRA KO samples are colored as indicated, and PERMANOVA statistics are shown in each panel. (**B**) Volcano plots of differentially abundant colonic mucosal metabolite features in negative-ion mode and positive-ion mode. The x-axis shows log_2_ fold change in DRA KO versus WT, and the y-axis shows −log_10_ FDR. Numbers of significantly decreased and increased features in DRA KO are indicated, and representative metabolite features are labeled. (**C**) Top metabolites ranked by variable importance in projection (VIP) scores from PLS-DA among MSI level 1 annotated metabolites in negative-ion and positive-ion modes. The dashed line indicates VIP = 1.5. Bar color indicates direction of change in DRA KO versus WT, and colored metabolite labels indicate features that also reached FDR significance. PLS-DA/VIP scores were used for feature prioritization and visualization only; differential abundance was defined by univariate testing with FDR correction and fold-change criteria, and VIP score alone was not used to define significance. (**D**) Pathway topology analysis of altered colonic mucosal metabolites across four complementary input layers: Layer 1, all significant annotated metabolites; Layer 2, significant MSI level 1 metabolites; Layer 3, VIP-prioritized MSI level 1 metabolites; and Layer 4, VIP-prioritized MSI level 3 metabolites. Dot size indicates pathway impact, dot color indicates −log_10_ FDR, and FDR class indicates pathways reaching FDR < 0.05 or suggestive FDR 0.05–0.10. (**E**) Shared KEGG pathways identified from colonic transcriptomic and metabolomic analyses. Columns show pathway evidence from MSI level 1 metabolite-based pathway analysis, RNA-seq significant genes, and combined transcriptomic-metabolomic pathway evidence. Dot size indicates the number of matched features, and color indicates −log_10_ FDR. Because the colonic RNA-seq and metabolomics datasets were generated from independent mouse cohorts, the shared pathways shown in panel e represent pathway-level convergence rather than sample-level gene-metabolite correlations within the same animals. Shared pathways include arginine biosynthesis, glutathione metabolism, lysine degradation, arginine and proline metabolism, and histidine metabolism. (**F**,**G**) Pathway-level maps for representative shared pathways, including arginine biosynthesis and glutathione metabolism. Triangles denote genes and squares denote metabolites. Node color indicates log_2_ fold change in DRA KO versus WT. Abbreviations: WT, wild-type; KO, knockout; DRA, down-regulated in adenoma; LC–MS, liquid chromatography-mass spectrometry; PERMANOVA, permutational multivariate analysis of variance; FDR, false discovery rate; PLS-DA, partial least-squares discriminant analysis; VIP, variable importance in projection; MSI, Metabolomics Standards Initiative; KEGG, Kyoto Encyclopedia of Genes and Genomes; BCAA, branched-chain amino acid; NAM, nicotinamide; CoA, coenzyme A; Gly/Ser/Thr, glycine/serine/threonine metabolism; Ala/Asp/Glu, alanine/aspartate/glutamate metabolism; Arg/Pro, arginine/proline metabolism; Phe/Tyr/Trp, phenylalanine/tyrosine/tryptophan; Arg biosyn., arginine biosynthesis; Lysine degr., lysine degradation.

Building on the human DRA-associated metabolic signature and the metabolism-focused transcriptional remodeling observed in DRA KO colon, we next asked whether DRA deficiency was accompanied by corresponding changes in the colonic mucosal metabolome. Untargeted LC-MS metabolomic profiling was performed in an independent mouse cohort with n = 5 biological replicates per genotype and showed genotype-associated separation between WT and DRA KO colonic mucosal samples in both negative- and positive-ion modes (Figure 3A). Univariate analysis identified significant metabolite features in both modes, including 31 increased and 8 decreased features in negative mode and 75 increased and 12 decreased features in positive mode after FDR correction (Figure 3B). Since the number of confidently annotated metabolites was more limited than the transcriptomic gene set, we emphasized pathway-level interpretation rather than overinterpreting individual metabolite features.

We next examined MSI level 1 metabolites prioritized by variable importance in projection (VIP) scores from the PLS-DA model to identify high-confidence features contributing to genotype separation (Figure 3C). VIP-ranked metabolites were used for feature prioritization and were interpreted together with univariate FDR and fold-change criteria, annotation confidence, and pathway context. These metabolites mapped broadly to amino acid/nitrogen metabolism, redox and cofactor pathways, nucleotide metabolism, lipid-related metabolism, and host-microbial metabolic products. VIP-prioritized MSI level 3 features were evaluated separately as exploratory support and included putative signals related to glycan/mucin-associated metabolism, glutathione/redox metabolism, amino acid/nitrogen metabolism, nucleotide metabolism, and short-chain organic acid-related metabolism (Supplementary Figure S3).

To determine whether altered metabolites converged on shared pathways, we used four complementary pathway-analysis layers that differed by statistical filtering and annotation confidence: all significant annotated metabolites, significant MSI level 1 metabolites, VIP-prioritized MSI level 1 metabolites, and VIP-prioritized MSI level 3 metabolites (Figure 3D). Interpretation was anchored primarily in the statistically significant annotated metabolite layers, particularly MSI level 1, whereas VIP-prioritized MSI level 3 features were considered exploratory support. Arginine biosynthesis emerged as the most consistent pathway, appearing across all four layers and reaching significance in both statistically significant metabolite-based layers and the MSI level 1 VIP-prioritized layer.

We next compared the independent transcriptomic and metabolomic results at the pathway level to determine whether the metabolite changes aligned with the metabolic transcriptional remodeling observed in DRA KO colon. For this comparison, metabolite evidence was anchored in the significant MSI level 1 metabolite layer to prioritize high-confidence annotated metabolites. Several pathways were shared between the transcriptomic and metabolomic results, including arginine biosynthesis, glutathione metabolism, lysine degradation, arginine and proline metabolism, and histidine metabolism (Figure 3E). We then mapped gene and metabolite changes onto representative shared pathways, highlighting convergent alterations in arginine biosynthesis and glutathione metabolism (Figure 3F,G). Together, these findings show that DRA deficiency is associated with overlapping pathway-level transcriptomic and metabolomic changes in amino acid/nitrogen and redox-related metabolism.

### 3.4. DRA Deficiency Is Associated with Fecal Metabolome Remodeling Enriched for Amino Acid and Polyamine-Related Pathways

**Figure 4:**
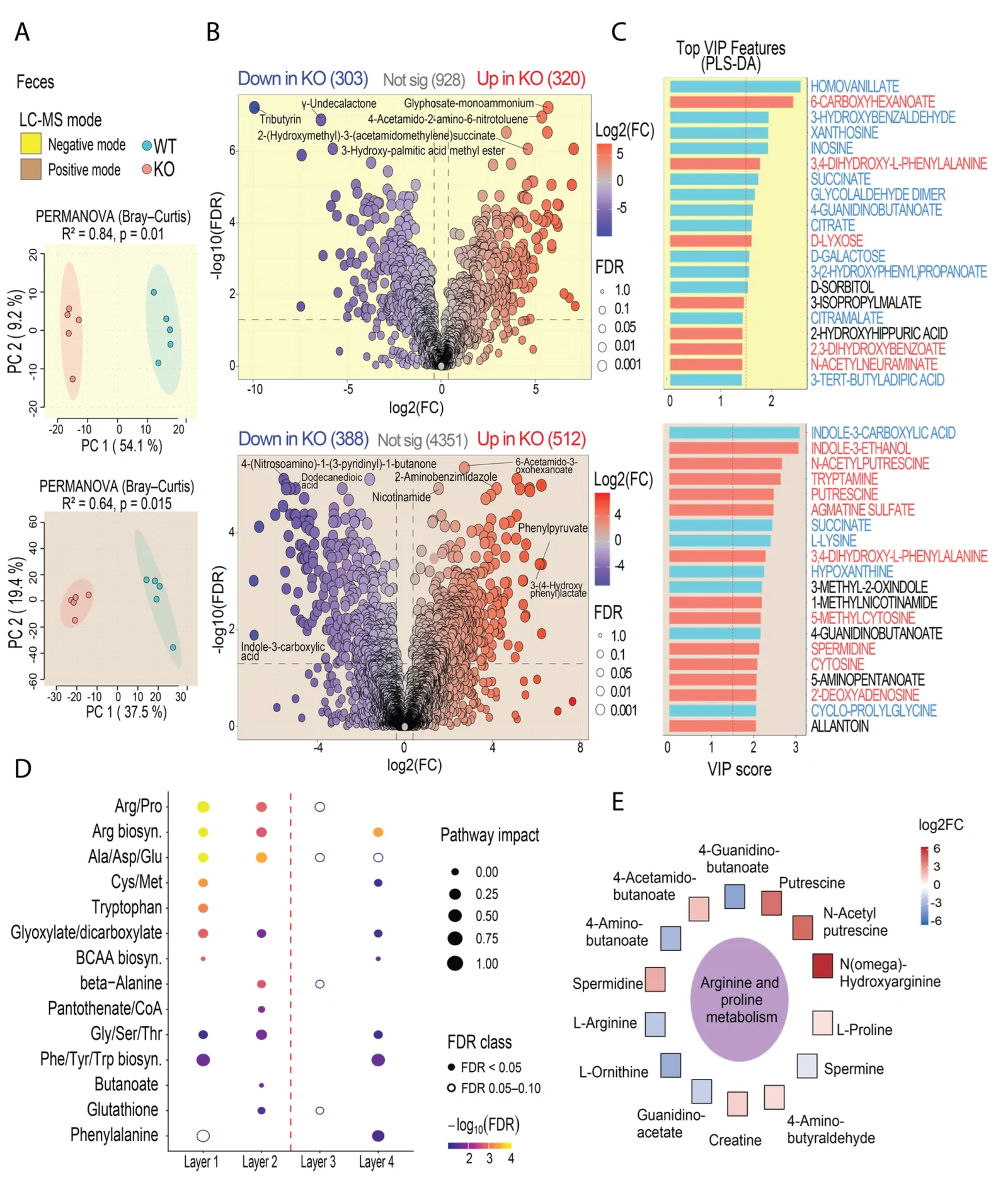
**DRA deficiency is associated with fecal metabolome remodeling enriched for amino acid and polyamine-related pathways.** Untargeted LC–MS metabolomics was performed on fecal samples from a separate WT and DRA KO mouse cohort with n = 5 biological replicates per genotype in negative- and positive-ion modes. (**A**) Principal component analysis of fecal metabolites acquired in negative-ion mode and positive-ion mode. Each point represents one biological replicate. WT and DRA KO samples are colored as indicated, and PERMANOVA statistics are shown in each panel. (**B**) Volcano plots of differentially abundant fecal metabolite features in negative-ion mode and positive-ion mode. The x-axis shows log_2_ fold change in DRA KO versus WT, and the y-axis shows −log_10_ FDR. Numbers of significantly decreased and increased features in DRA KO are indicated, and representative metabolite features are labeled. (**C**) Top variable importance in projection (VIP)-ranked MSI level 1 fecal metabolites identified by partial least-squares discriminant analysis (PLS-DA). The dashed line indicates the VIP = 1.5 threshold used for prioritization. Bar color indicates direction of change in DRA KO versus WT, with red indicating higher abundance in DRA KO and blue indicating lower abundance in DRA KO. Non-black metabolite labels indicate features that also reached FDR significance, with label color following the same direction as the bar. PLS-DA/VIP scores were used for feature prioritization and visualization only; differential abundance was defined by univariate testing with FDR correction and fold-change criteria, and VIP score alone was not used to define significance. (**D**) Pathway topology analysis of altered fecal metabolites across four complementary input layers: **Layer 1**, all significant annotated metabolites; **Layer 2**, significant MSI level 1 metabolites; **Layer 3**, VIP-prioritized MSI level 1 metabolites; and **Layer 4**, VIP-prioritized MSI level 3 metabolites. Dot size indicates pathway impact, dot color indicates −log_10_ FDR, and FDR class indicates pathways reaching FDR < 0.05 or suggestive FDR 0.05–0.10. (**E**) Arginine and proline metabolism pathway map showing altered fecal metabolites in DRA KO mice. Metabolites are colored according to log_2_ fold change in DRA KO versus WT, highlighting polyamine-related metabolites, arginine- and ornithine-related intermediates, and guanidino/creatine-related metabolites. **Abbreviations:** DRA, down-regulated in adenoma WT, wild-type; KO, knockout; LC–MS, liquid chromatography-mass spectrometry; PERMANOVA, permutational multivariate analysis of variance; FDR, false discovery rate; FC, fold change; PLS-DA, partial least-squares discriminant analysis; VIP, variable importance in projection; MSI, Metabolomics Standards Initiative; BCAA, branched-chain amino acid; CoA, coenzyme A; Arg/Pro, arginine/proline metabolism; Arg biosyn., arginine biosynthesis; Ala/Asp/Glu, alanine/aspartate/glutamate metabolism; Cys/Met, cysteine/methionine metabolism; Gly/Ser/Thr, glycine/serine/threonine metabolism; Phe/Tyr/Trp, phenylalanine/tyrosine/tryptophan biosynthesis.

Given the apical localization of DRA and its potential role in shaping the intestinal luminal environment, we next examined whether DRA deficiency was accompanied by changes in the fecal metabolome. Fecal untargeted LC-MS metabolomics was performed using the same metabolomics-associated cohort with n = 5 biological replicates per genotype. Fecal metabolomic profiles showed clear genotype-associated separation between WT and DRA KO mice in both ion modes (Figure 4A). Consistent with this separation, hundreds of fecal metabolite features differed between WT and DRA KO mice after FDR correction (Figure 4B), supporting a marked shift in the luminal metabolic profile.

We next examined VIP-prioritized MSI level 1 fecal metabolites contributing to genotype separation (Figure 4C). These high-confidence annotated features included metabolites related to amino acid and polyamine metabolism, nucleotide metabolism, organic acids, host-microbial metabolites, and lipid-associated metabolism. VIP-prioritized MSI level 3 features were evaluated separately and included putative signals related to amino acid/nitrogen, aromatic amino acid-derived host-microbial, short-chain/organic acid, and lipid-associated metabolism (Supplementary Figure S4).

We then asked whether the fecal metabolite changes converged on common metabolic pathways using the same four-layer pathway-analysis framework applied to the colonic mucosal metabolome. Similar to the mucosal metabolome, fecal pathway analysis highlighted amino acid, nitrogen, redox, and cofactor-related metabolism (Figure 4D). However, the fecal profile showed a more prominent luminal amino acid signal, with several amino acid-related pathways appearing in the statistically significant metabolite layers rather than only in VIP-prioritized or exploratory layers. The fecal arginine and proline metabolism map further showed that this signal involved polyamine metabolism, including putrescine-related metabolites, spermidine, spermine, and guanidino/creatine-related intermediates (Figure 4E). Together, these findings show that DRA deficiency is associated with fecal metabolomic remodeling that overlaps with the mucosal metabolic signature but is especially enriched for luminal amino acid and polyamine-related metabolism.

### 3.5. DRA Deficiency Is Associated with Systemic Serum Metabolomic Changes and Altered Bile Acid Profiles

**Figure 5:**
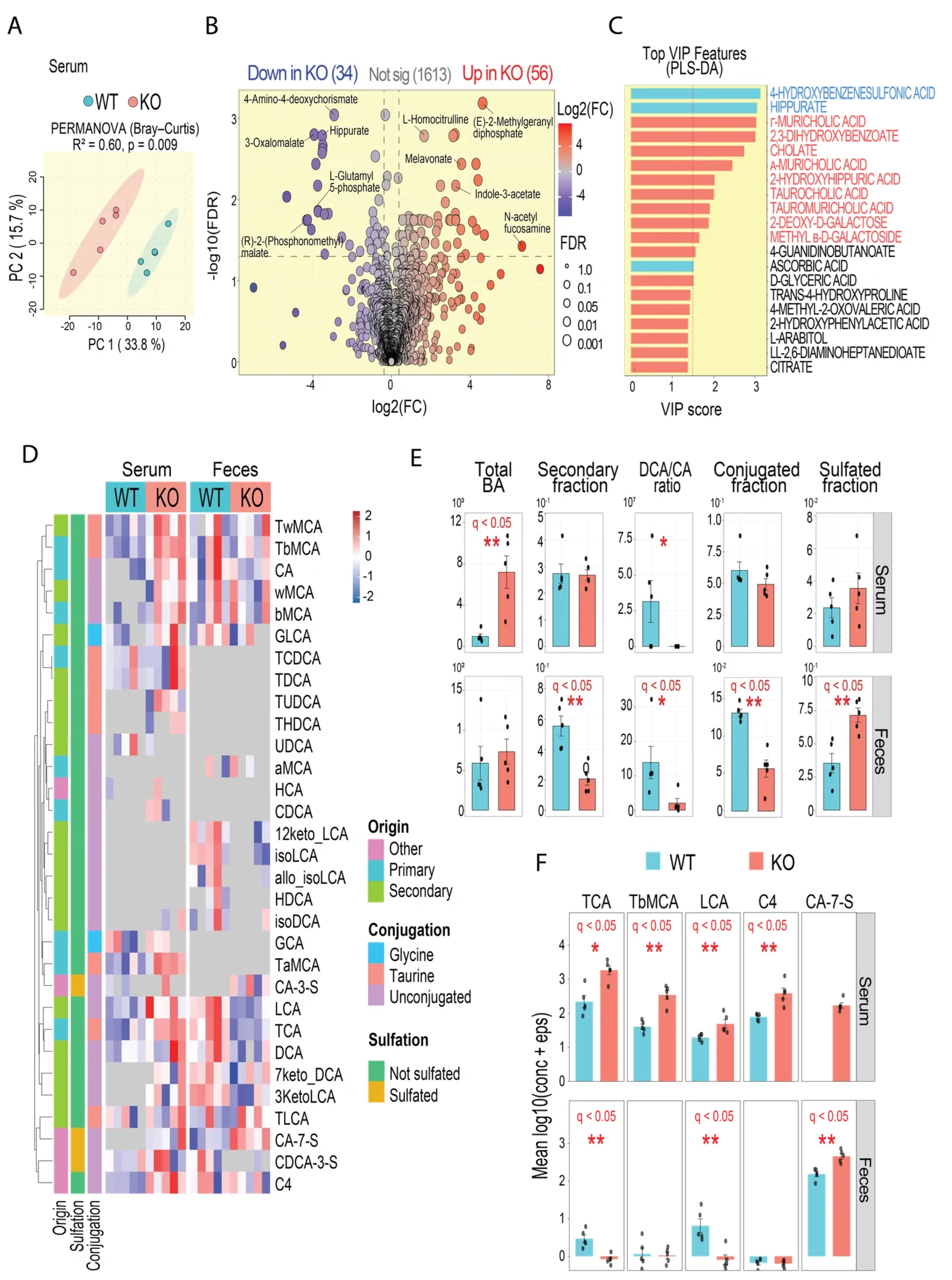
**DRA deficiency is associated with systemic serum metabolomic changes and altered bile acid profiles.** Untargeted serum LC-MS metabolomics was performed using a separate WT and DRA KO mouse cohort with n = 5 biological replicates per genotype. Targeted bile acid profiling of serum and feces was performed using a separate WT and DRA KO mouse cohort with n = 5 biological replicates per genotype. (**A**) Principal component analysis of serum metabolites acquired in negative-ion mode. Each point represents one biological replicate. WT and DRA KO samples are colored as indicated, and PERMANOVA statistics are shown. (**B**) Volcano plot of differentially abundant serum metabolite features in negative-ion mode. The x-axis shows log_2_ fold change in DRA KO versus WT, and the y-axis shows −log_10_ FDR. Numbers of significantly decreased and increased features in DRA KO are indicated, and representative metabolite features are labeled. (**C**) Top variable importance in projection (VIP)-ranked MSI level 1 serum metabolites identified by partial least-squares discriminant analysis (PLS-DA) in negative-ion mode. The dashed line indicates the VIP = 1.5 threshold used for prioritization. Bar color indicates direction of change in DRA KO versus WT, with red indicating higher abundance in DRA KO and blue indicating lower abundance in DRA KO. Non-black metabolite labels indicate features that also reached FDR significance, with label color following the same direction as the bar. PLS-DA/VIP scores were used for feature prioritization and visualization only; differential abundance was defined by univariate testing with FDR correction and fold-change criteria, and VIP score alone was not used to define significance. (**D**) Heatmap of targeted bile acid profiles in serum and feces from WT and DRA KO mice. Rows represent bile acid species or the bile acid synthesis marker C4, and columns represent biological replicates. Row annotations indicate bile acid origin, conjugation status, and sulfation status. Values are shown as row-scaled z scores within each compartment. (**E**) Summary bile acid metrics in serum and feces, including total bile acids, secondary bile acid fraction, deoxycholic acid/cholic acid ratio, conjugated bile acid fraction, and sulfated bile acid fraction. Bars show mean ± SEM with individual biological replicates overlaid (n = 5 mice per genotype). (**F**) Representative bile acid species and bile acid synthesis marker measurements in serum and feces, including taurocholic acid, tauro-beta-muricholic acid, lithocholic acid, C4, and cholic acid 7-sulfate. Bars show group summaries with individual biological replicates overlaid. For panels (**E**,**F**), WT and DRA KO groups were compared separately within serum and feces using Wilcoxon rank-sum tests. Asterisks denote nominal, unadjusted *p* values: * p < 0.05 and ** p < 0.01. q values indicate Benjamini-Hochberg FDR-adjusted *p* values calculated within each bile acid comparison family and compartment. Findings were considered significant after multiple-testing correction only when q < 0.05. **Abbreviations:** DRA, down-regulated in adenoma; WT, wild-type; KO, knockout; LC–MS, liquid chromatography-mass spectrometry; PERMANOVA, permutational multivariate analysis of variance; FDR, false discovery rate; FC, fold change; VIP, variable importance in projection; MSI, Metabolomics Standards Initiative; PLS-DA, partial least-squares discriminant analysis; BA, bile acid; DCA, deoxycholic acid; CA, cholic acid; TCA, taurocholic acid; TbMCA, tauro-beta-muricholic acid; LCA, lithocholic acid; CA-7-S, cholic acid 7-sulfate; C4, 7α-hydroxy-4-cholesten-3-one.

After identifying DRA-associated metabolic remodeling in colonic mucosa and feces, we next examined whether DRA deficiency was accompanied by systemic metabolic changes. Serum untargeted LC-MS metabolomics was performed using a separate WT and DRA KO mouse cohort with n = 5 biological replicates per genotype and showed genotype-associated separation between WT and DRA KO mice in negative-ion mode, with a similar separation pattern observed in positive-ion mode in the supplementary analysis (Figure 5A; Supplementary Figure S5A). Consistent with this separation, serum metabolite features differed between WT and DRA KO mice after FDR correction, including 56 increased and 34 decreased features in negative-ion mode and 50 increased and 25 decreased features in positive-ion mode (Figure 5B; Supplementary Figure S5B).

VIP-prioritized MSI level 1 serum metabolites contributing to genotype separation included bile acid-related metabolites in both ion modes, including muricholic acid species, tauromuricholic acid, taurocholic acid, cholate, and 7-ketochenodeoxycholic acid (Figure 5C; Supplementary Figure S5C). VIP-prioritized MSI level 3 serum features provided exploratory support for additional bile acid/sterol, amino acid, host-microbial, and organic acid-related signals (Supplementary Figure S5D,E). Compared with the colonic mucosal and fecal metabolomes, serum pathway-level signals were more modest and were observed mainly in the VIP-prioritized MSI level 3 layer (Supplementary Figure S5F).

Since the high-confidence untargeted serum findings prominently included bile acid-related features, we next used targeted bile acid profiling to quantify defined bile acid species and determine whether this signal extended across serum and feces. Targeted profiling supported compartment-specific bile acid remodeling in DRA KO mice. In serum, DRA deficiency was associated with altered total bile acid burden and changes in selected bile acid species, whereas the fecal profile showed stronger compositional remodeling involving secondary bile acids, the deoxycholic acid/cholic acid (DCA/CA) ratio, conjugated bile acids, and sulfated bile acids (Figure 5D,E; Supplementary Figure S5G). Representative measurements, including taurocholic acid (TCA), tauro-beta-muricholic acid (TbMCA), lithocholic acid (LCA), cholic acid 7-sulfate (CA-7-S), and the bile acid synthesis marker 7α-hydroxy-4-cholesten-3-one (C4), further illustrated these serum- and feces-specific patterns (Figure 5F). Together, these findings indicate that the bile acid-related signal detected in untargeted serum metabolomics was accompanied by broader systemic and luminal bile acid remodeling in DRA KO mice.

### 3.6. MetOrigin Annotation Highlights Host–Microbiota Co-Metabolism as a Major Feature of DRA Deficiency-Associated Metabolic Remodeling

**Figure 6:**
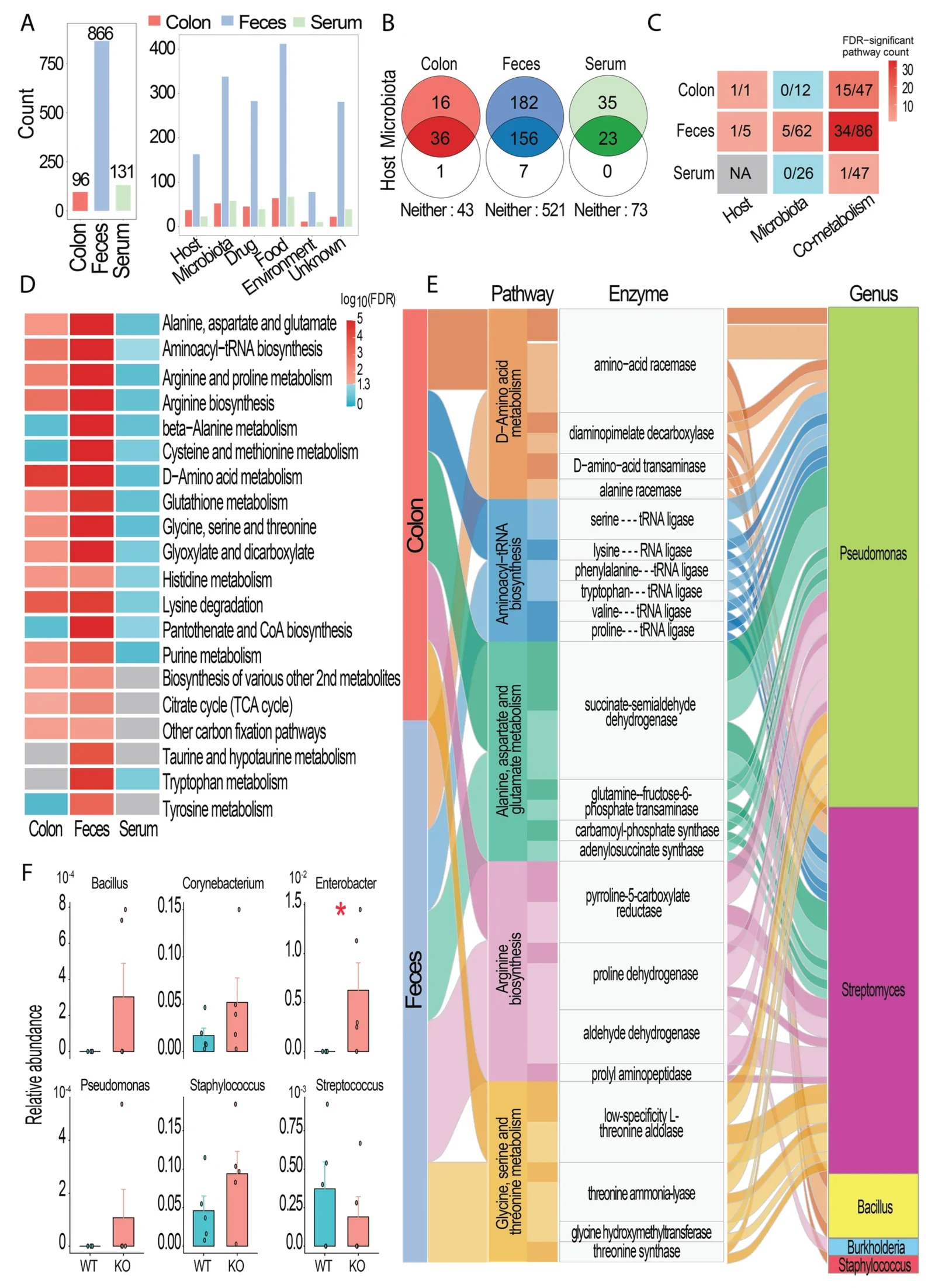
**MetOrigin annotation highlights inferred host-microbiota co-metabolism features across compartments.** MetOrigin analysis was performed using altered metabolite features from the untargeted LC-MS metabolomics datasets generated with n = 5 biological replicates per genotype. Fecal 16S rRNA sequencing was performed using a separate WT and DRA KO mouse cohort with n = 5 biological replicates per genotype. MetOrigin assignments were treated as computational source-attribution annotations rather than definitive metabolite origins. (**A**) Counts of altered metabolite features for colon, feces, and serum, defined as FDR-significant features from volcano analysis after combining and deduplicating positive- and negative-ion mode results, together with MetOrigin source-attribution annotations of altered features. Features were assigned to host-, microbiota-, drug-, food-, environment-associated, or unknown categories. Categories are not mutually exclusive. Colors denote compartment: colon, red; feces, blue; serum, green. (**B**) Venn diagrams showing overlap of MetOrigin source assignments among host- and microbiota-associated categories within each compartment. “Neither” indicates features not assigned to host or microbiota categories. (**C**) Fraction of pathways identified within each origin class and compartment, shown as the number of FDR-significant pathways over the total number of detected pathways. Color intensity reflects the number of FDR-significant pathways; cyan indicates no pathways passing FDR correction, and gray indicates not detected. (**D**) Top inferred host-microbiota co-metabolism pathways across colon, feces, and serum. Pathways passing FDR correction are shown in red, with intensity reflecting significance; cyan indicates pathways tested but not significant after FDR correction, and gray indicates undetected pathways. (**E**) Alluvial plot showing the top five host-microbiota co-metabolism pathways in colon and feces linked to implicated microbial enzymes and associated genera. (**F**) Relative abundance of selected genera represented among the top co-metabolism pathways based on fecal 16S rRNA sequencing data. These genera were evaluated descriptively because they were represented in the MetOrigin co-metabolism framework. WT is shown in blue and DRA KO in red. Asterisks denote raw Wilcoxon rank-sum test significance (* *p* < 0.05). **Abbreviations:** DRA, down-regulated in adenoma; WT, wild-type; KO, knockout; LC–MS, liquid chromatography–mass spectrometry; FDR, false discovery rate; 16S rRNA, 16S ribosomal RNA.

To further interpret the compartment-specific metabolomic changes, we applied MetOrigin annotation to altered metabolite features from colon, feces, and serum. Fecal 16S rRNA profiling was performed using a separate WT and DRA KO mouse cohort with n = 5 biological replicates per genotype and was used descriptively, rather than as a comprehensive microbiome analysis, to evaluate genera represented in the MetOrigin co-metabolism framework. MetOrigin distributed altered features across host-, microbiota-, food-, drug-, environment-associated, and unknown categories, which were treated as non-mutually exclusive source-attribution annotations rather than definitive metabolite origins (Figure 6A). When host and microbiota-associated assignments were examined directly, colon and feces showed substantial overlap, supporting inferred host-microbiota co-metabolism as a prominent inferred feature of the DRA-associated metabolite signature (Figure 6B).

At the pathway level, MetOrigin-inferred host-microbiota co-metabolism accounted for the largest number of FDR-significant enrichments, particularly in feces and colon, whereas host-only and microbiota-only pathway assignments contributed less prominently (Figure 6C). These co-metabolic enrichments clustered around amino acid biosynthesis and interconversion, nitrogen-linked amino acid metabolism, carbon metabolism, and redox-associated glutathione metabolism (Figure 6D).

We next examined the pathway-enzyme-genus structure of these inferred co-metabolic annotations. In colon and feces, selected co-metabolism pathways were linked to enzymes involved in amino acid interconversion, tRNA ligation, transamination, and related biosynthetic reactions, with associated genera including *Pseudomonas*, *Streptomyces*, *Bacillus*, *Burkholderia*, and* Staphylococcus* (Figure 6E). The expanded alluvial analysis showed that this pathway-enzyme-genus structure extended across additional co-metabolism pathways in colon and feces (Supplementary Figure S6). Descriptive comparison with fecal 16S rRNA profiling showed that selected genera represented in the MetOrigin framework were detectable, with Enterobacter showing a significant genotype-associated difference and other genera showing directional shifts in DRA KO mice (Figure 6F). Together, these findings support inferred host-microbiota co-metabolism, especially amino acid-related co-metabolism in fecal and colonic compartments, as a major organizing feature of DRA deficiency-associated metabolic remodeling.

## 4. Discussion

DRA/SLC26A3 is a major apical Cl^-^/HCO_3_^-^ exchanger in the colon, with roles in fluid absorption, epithelial barrier integrity [12], bicarbonate-dependent mucus organization [13], microbial maintenance, antimicrobial peptide levels [14], and epithelial–immune interactions [15]. The present study extends this framework by linking reduced DRA expression or genetic DRA deficiency to metabolic remodeling across mucosal, luminal, and systemic compartments. Rather than positioning DRA as a direct regulator of isolated metabolic enzymes, our findings support a broader model in which altered DRA status is associated with disruption of the epithelial–luminal microenvironment, where ion transport, barrier function, microbial ecology, and host–microbe metabolism are interconnected. Importantly, many metabolic alterations identified here overlap with broader metabolic features previously described in UC/IBD and should not be interpreted as DRA-specific metabolic changes.

Human UC has been widely characterized by metabolic disruption, including changes in amino acid metabolism [39,40,41,42,43,44], redox balance [45,46,47], and bile acid signaling [48,49,50]. Our cross-cohort analysis builds on this background by showing that many UC-altered metabolic pathways also varied with DRA expression levels among UC samples. Since these human pathway scores were inferred from bulk transcriptomic data using GSVA/ssGSEA, they should be interpreted as inferred transcriptional activity of metabolic pathways rather than direct measurements of metabolic flux, metabolite abundance, or biochemical enzyme activity. Most pathways with lower inferred transcriptional activity in UC were positively associated with DRA expression, suggesting that higher DRA levels marked greater preservation of metabolism-associated transcriptional programs, whereas lower DRA levels were associated with greater suppression of these inferred pathway scores. In addition to this broad inferred transcriptional suppression of metabolism-associated pathways, a smaller set of glycan- and mucosal structure-related pathways was increased in UC and also varied with DRA expression, suggesting that low-DRA UC mucosa captures both metabolic suppression and mucosal glycan/barrier remodeling. This interpretation is consistent with disturbed mucus-barrier architecture in UC, including early structural weakening of the colonic mucus barrier [51], and with the loss of the firmly adherent mucus layer reported in *Slc26a3*-deficient mice [13]. Because both DRA expression and pathway-level signals were derived from bulk transcriptomic profiles of human colonic mucosal biopsies, these analyses cannot identify the exact cell type or mechanism responsible. Bulk mucosal DRA transcript abundance may reflect reduced epithelial DRA expression, altered epithelial composition, inflammatory cell infiltration, or a combination of these factors. Even with this limitation, the recurrence of these associations across cohorts supports reduced mucosal DRA expression as part of a reproducible metabolically and structurally altered UC mucosal state, consistent with genetic and clinical evidence linking SLC26A3/DRA dysfunction to UC susceptibility and IBD risk [8,9,10,11].

The DRA KO mouse model strengthened the human findings by showing that DRA deficiency is accompanied by metabolic disruption in an experimental setting. In the colon, the transcriptomic analysis showed broad reorganization across energy/redox, central carbon, lipid, xenobiotic, cofactor, and amino acid pathways, whereas the mucosal metabolome highlighted a more focused amino acid- and redox-related signature. These mucosal metabolomic interpretations were anchored primarily in statistically significant annotated metabolites and MSI level 1 features, whereas MSI level 3 features were used as exploratory support. The strongest transcriptome–metabolome pathway overlap involved arginine biosynthesis, arginine/proline metabolism, histidine metabolism, lysine degradation, and glutathione metabolism. Notably, these pathways overlap with recurrent themes in UC and intestinal health, including amino acid metabolism [39,40,41], host-microbiome metabolic networks [40], arginine availability [42,43,44], and glutathione/redox balance [45,46,47]. Although the transcriptomic and metabolomic datasets came from independent mouse cohorts and cannot define sample-level gene–metabolite coupling within the same animals, their pathway-level convergence suggests that DRA deficiency is associated with a less homeostatic colonic mucosal metabolic state that could affect mucosal resilience in inflammatory settings. However, the present study does not establish that these metabolic changes cause or mediate inflammatory susceptibility.

A possible link between DRA deficiency and metabolic remodeling is disruption of the epithelial surface environment. DRA loss is associated with reduced bicarbonate secretion and impaired mucus organization [6,13], as well as changes in epithelial barrier function and microbial ecology [12,14]. Together with altered luminal pH, fluid movement, and transit, these changes may reshape the mucosal–luminal niche where nutrients, microbial metabolites, and epithelial stress signals interact. Altered amino acid availability could affect epithelial cells through nutrient-sensing and stress-response pathways, including GCN2-mediated amino acid sensing [52] and integrated stress signaling during amino acid deprivation [53]. Changes in metabolic intermediates may influence chromatin regulation [54,55], while ER stress can further contribute to epithelial dysfunction and intestinal inflammation [56,57,58]. These mechanisms were not directly tested here, and the global DRA KO model cannot separate epithelial-intrinsic effects from secondary consequences of diarrhea, altered luminal pH, increased fecal water content, altered transit, inflammation, or dysbiosis. In addition, all animals, including both WT and DRA KO mice, received oral electrolyte solution to reduce dehydration risk in DRA KO mice and to avoid comparing dehydrated DRA KO animals with normally hydrated WT controls. Thus, electrolyte exposure was not genotype-specific. However, because DRA KO mice have altered intestinal fluid and electrolyte handling, we cannot exclude the possibility that electrolyte supplementation interacted with genotype-dependent physiology to influence luminal, mucosal, or systemic metabolic profiles. Therefore, the data are best interpreted as genotype-associated metabolic differences under standardized electrolyte-supported conditions and as a combined epithelial–luminal disturbance, rather than as a single linear pathway from DRA loss to metabolism. The inability to fully disentangle these effects represents one of the few limitations of the study.

The fecal, MetOrigin, and bile acid findings provide further support for this mucosal-luminal niche model. Feces showed broader metabolite changes than colonic tissue, with prominent amino acid and polyamine-related signals suggesting remodeling of the luminal biochemical environment. Since fecal metabolites can arise from epithelial responses, microbial metabolism, diet, absorption, or altered transit, they should not be assigned to a single source. MetOrigin linked many altered colon and fecal metabolites to inferred host–microbiota co-metabolic pathways, particularly amino acid-related pathways. However, these computational source-attribution assignments do not directly establish metabolite origin or metabolic flux [33].

Bile acids provided a complementary example of altered host and microbial chemistry across compartments. Serum and feces showed distinct patterns, suggesting changes in circulating bile acid burden, luminal bile acid composition, and bile acid processing. This is biologically relevant because bile acids reflect host synthesis and enterohepatic circulation [48], microbial transformation in the gut lumen [49], and signaling pathways implicated in IBD [48,50]. The reduced secondary bile acid fraction and altered deoxycholic acid/cholic acid ratio may reflect altered microbial conversion, whereas increased serum C4 and altered sulfated bile acids may reflect changes in bile acid synthesis, feedback, detoxification, or elimination [59,60,61]. These mechanisms are not mutually exclusive and require direct testing.

Similar epithelial-luminal disturbances have been reported in NHE3 [62,63,64] and PAT1/SLC26A6 [65] deficiency models, which have been linked to dysbiosis, barrier dysfunction, altered microbiome-associated metabolites, and increased susceptibility to intestinal inflammation. However, the present study does not establish that the metabolic changes identified here cause or mediate inflammatory susceptibility. Future studies using inducible or epithelial-specific DRA models, paired multi-omics, organoid–microbiota systems, microbiota manipulation, and isotope tracing will be needed to determine whether the metabolic changes identified here contribute causally to inflammatory susceptibility.

## 5. Conclusions

Overall, our findings suggest that reduced DRA/SLC26A3 is linked to a metabolically vulnerable intestinal state. In human UC, reduced mucosal DRA expression was associated with widespread suppression of inferred metabolic pathway activity. In DRA-deficient mice, metabolic alterations extended across colonic tissue, feces, and serum, involving amino acid, redox, polyamine, bile acid, and inferred host–microbiota co-metabolic signatures. Together, these findings suggest that loss of DRA function is associated with reduced metabolic stability at the mucosal–luminal interface, defining a potentially inflammation-relevant state of the epithelial–luminal axis that may increase susceptibility to intestinal inflammation.

## Data Availability

Raw RNA-seq FASTQ files are available in the NCBI Sequence Read Archive under BioProject PRJNA1345427. Untargeted LC–MS metabolomics data are available in GNPS/MassIVE under accession numbers MSV000099473 for positive-ion mode and MSV000099476 for negative-ion mode. Detailed experimental protocols, analysis parameters, workflow descriptions, and derived summary tables are available at Zenodo under DOI: https://doi.org/10.5281/zenodo.21704235.

## Supplementary Materials

The following supporting information can be downloaded at: https://www.mdpi.com/article/10.3390/metabo16090690/s1, Supplementary Methods: Detailed materials and methods, including expanded experimental protocols, analysis parameters, and workflow descriptions; Figure S1: Expanded cross-cohort pathway and DRA meta-analysis; Figure S2: Full KEGG metabolism pathway-score correlation heatmap; Figure S3: VIP-prioritized putatively annotated metabolite features in DRA KO colonic mucosa; Figure S4: VIP-prioritized putatively annotated metabolite features in DRA KO feces; Figure S5: Additional serum metabolomic analyses and expanded bile acid metric summaries; Figure S6: Expanded alluvial plot of the top 15 host-microbiota co-metabolism pathways in colon and feces linked to microbial enzymes and associated genera.

## Abbreviations

The following abbreviations are used in this manuscript:

16S rRNA	16S ribosomal RNA
AI	artificial intelligence
Ala/Asp/Glu	alanine, aspartate, and glutamate metabolism
Arg biosyn.	arginine biosynthesis
Arg/Pro	arginine and proline metabolism
BA	bile acid
BCAA	branched-chain amino acids
biosyn.	biosynthesis
bp	base pair
C4	7α-hydroxy-4-cholesten-3-one
CA	cholic acid
CA-7-S	cholic acid 7-sulfate
CI	confidence interval
CLD	congenital chloride diarrhea
CoA	coenzyme A
CS/DS	chondroitin sulfate/dermatan sulfate
CYP	cytochrome P450
Cys/Met	cysteine and methionine metabolism
DADA2	Divisive Amplicon Denoising Algorithm 2
DCA	deoxycholic acid
DEG	differentially expressed gene
degr.	degradation
DNase	deoxyribonuclease
DOI	digital object identifier
DRA	down-regulated in adenoma
DRA meta	random-effects pooled DRA–pathway association across UC samples
Drug-CYP	drug metabolism by cytochrome P450
Drug metab.	drug metabolism
ER	endoplasmic reticulum
ESI	electrospray ionization
FA	fatty acid
FC	fold change
FDR	false discovery rate
GAG	glycosaminoglycan
GCN2	general control nonderepressible 2
Gluconeo.	gluconeogenesis
Gly/Ser/Thr	glycine, serine, and threonine metabolism
GNPS	Global Natural Products Social Molecular Networking
GPI	glycosylphosphatidylinositol
GSEA	gene set enrichment analysis
GSVA	gene set variation analysis
H	healthy/control
HS	heparan sulfate
IACUC	Institutional Animal Care and Use Committee
IBD	inflammatory bowel disease
KEGG	Kyoto Encyclopedia of Genes and Genomes
KO	knockout
KO-down	decreased in DRA-knockout versus wild-type mice
KO-up	increased in DRA-knockout versus wild-type mice
LCA	lithocholic acid
LC–MS	liquid chromatography–mass spectrometry
LC–MS/MS	liquid chromatography–tandem mass spectrometry
Lysine degr.	lysine degradation
MassIVE	Mass Spectrometry Interactive Virtual Environment
metab.	metabolism
mRNA	messenger RNA
MSI	Metabolomics Standards Initiative
NAM	nicotinamide
NCBI	National Center for Biotechnology Information
NHE3	Na^+^/H^+^ exchanger 3 (SLC9A3)
OXPHOS	oxidative phosphorylation
PAT1	putative anion transporter 1
PCA	principal component analysis
PC1 or 2	principal component 1 or 2
PERMANOVA	permutational multivariate analysis of variance
Phe/Tyr/Trp	phenylalanine, tyrosine, and tryptophan biosynthesis
PLS-DA	partial least-squares discriminant analysis
PPP	pentose phosphate pathway
QIIME	Quantitative Insights Into Microbial Ecology
RNA-seq	RNA sequencing
rRNA	ribosomal RNA
SLC26A3	solute carrier family 26 member 3
SLC26A6	solute carrier family 26 member 6
STAR	Spliced Transcripts Alignment to a Reference
TCA	taurocholic acid
TCA cycle	tricarboxylic acid cycle
TbMCA	tauro-β-muricholic acid
UC	ulcerative colitis
UHPLC	ultrahigh-performance liquid chromatography
Unsat. FA	unsaturated fatty acid
VIP	variable importance in projection
WT	wild-type
Xenobiotic-CYP	xenobiotic metabolism by cytochrome P450
