# Supplementary material for: Multi-Compartment Metabolic Remodeling in DRA-Deficient Mice Overlaps with Ulcerative Colitis Metabolic Signatures
Source: Metabolites. 2026 Sep 18;16(9):690. doi: 10.3390/metabo16090690 (PMC13609582; doi:10.3390/metabo16090690)
Supplement: Supplementary file 1 [file metabolites-16-00690-s001.zip › File Repository/01_Detailed Methods_DRA_Metabolomics.pdf]

Multi-compartment Metabolic Remodeling in DRA-Deficient Mice Overlaps with Ulcerative Colitis Metabolic Signatures

Jayson M. Antonio[1], Shubha Priyamvada[1], Arivarasu N. Anbazhagan[1], Harris J[1], Nathan Calzadilla[2], Rengul C. Atalay[3], Reena Sethi[4], Seema Saksena[3], Ravinder K. Gill[3], Gokhan M. Mutlu[5], Waddah A. Alrefai[1,3], Ece A. Mutlu[1,3], Anoop Kumar[1,3*], and Pradeep K. Dudeja[1,3*]

[1] Division of Gastroenterology and Hepatology, Department of Medicine, University of Illinois Chicago, Chicago, IL, USA.
[2] Department of Surgery, Section of Plastic and Reconstructive Surgery, The University of Chicago, Chicago, IL, USA.
[3] Jesse Brown VA Medical Center, Chicago, IL, USA.
[4] Department of Biology, Virginia Commonwealth University, Richmond, VA, USA.
[5] Department of Medicine, Section of Pulmonary and Critical Care Medicine, The University of Chicago, Chicago, IL, USA.
* Co-corresponding authors

Materials and Methods

2.1 Study design and analysis overview

This study integrated human transcriptomic pathway analysis with mouse transcriptomic, metabolomic, bile acid, and microbiome-associated analyses to define metabolic signatures associated with reduced or absent DRA/SLC26A3. First, six publicly available human colonic transcriptomic cohorts were analyzed to identify KEGG metabolic pathways altered in ulcerative colitis (UC) and to determine whether these pathways were associated with continuous SLC26A3/DRA expression within UC samples. Second, colonic mucosal RNA-seq from wild-type (WT) and DRA knockout (DRA KO) mice was analyzed using a metabolism-focused KEGG pathway framework. Third, untargeted LC-MS metabolomics was performed on colonic mucosa, feces, and serum from WT and DRA KO mice. Fourth, targeted bile acid profiling was performed in serum and feces. Finally, MetOrigin and 16S rRNA-based genus-level analyses were used to infer possible host, microbial, and host-microbiota co-metabolic contributions to altered metabolite signatures.

For human transcriptomic analyses, all models were fitted within each cohort before cross-cohort integration. Raw expression values and raw pathway scores were not pooled across datasets because the cohorts differed in transcriptomic platform, preprocessing method, expression scale, and sample composition. Cross-cohort inference was therefore based on standardized cohort-level effect estimates and their standard errors.

This document reports the key computational parameters required to reproduce the analyses, including input datasets, sample inclusion rules, pathway libraries, model structures, normalization procedures, statistical thresholds, multiple-testing procedures, and software platforms. It also provides the detailed experimental protocols, analysis parameters, and workflow descriptions used for the human transcriptomic, mouse transcriptomic, metabolomic, bile acid, MetOrigin, and 16S microbiome analyses. Study-specific derived tables are provided as accompanying repository files where applicable.

2.2 Human UC transcriptomic cohorts and input curation

The primary human analysis included six publicly available colonic mucosal transcriptomic cohorts: GSE11223, GSE38713, GSE206171, GSE206285, GSE53306, and GSE59071. These cohorts were selected because they contained UC and healthy/control colonic mucosal profiles and retained detectable SLC26A3 expression.

For the primary UC-versus-healthy/control pathway analysis, the six cohorts contributed 1,101 colonic expression profiles before participant-level aggregation in GSE206171. These included 123 UC and 67 healthy/control profiles from GSE11223; 30 UC and 13 control profiles from GSE38713; 114 UC and 38 healthy sigmoid-colon biopsy profiles from GSE206171; 550 UC and 18 healthy/control profiles from GSE206285; 28 UC and 12 healthy/control profiles from GSE53306; and 97 UC and 11 healthy/control profiles from GSE59071. For GSE206171, paired sigmoid biopsies from the same participant were averaged to participant-level profiles before ssGSEA scoring and statistical modeling, yielding 59 UC and 19 healthy participant-level analysis units for model-based analyses.

Processed expression matrices and phenotype metadata were curated and locked before statistical analysis. Expression values were retained on each cohort's native processed scale. No raw expression values were merged across cohorts. Gene identifiers were standardized to gene symbols when required, and duplicated gene symbols were collapsed within each cohort before downstream analysis. SLC26A3 expression was extracted from the processed expression matrix and used as the transcriptomic measure of DRA expression. DRA expression was analyzed as a numeric variable rather than categorized into high- and low-expression groups.

For the primary UC-versus-healthy/control KEGG pathway analysis, GSE11223 included all eligible colonic samples from ascending, descending, and sigmoid colon, while terminal ileal samples were excluded. Colonic region and patient structure were accounted for in the cohort-level model. For the final DRA-focused expression validation and within-UC DRA-pathway analysis, GSE11223 was reanalyzed using the locked descending-colon subset to match the tissue region used in the companion DRA-focused transcriptomic validation. This final descending-colon subset contained 34 UC and 23 healthy/control samples. The all-colon GSE11223 DRA analysis was retained as a sensitivity comparison, whereas the descending-colon analysis was used for the final DRA-focused expression and within-UC pathway-association analyses.

2.3 Human KEGG metabolic pathway reference and ssGSEA scoring

The human metabolic pathway universe was defined using KEGG Homo sapiens metabolic subpathways linked to the hsa01100 "Metabolic pathways" reference map. Individual KEGG metabolic subpathways were analyzed rather than treating hsa01100 as a single global pathway. Pathways were retained when they contained at least 10 genes in the locked human metabolic reference and at least five retrievable genes in each of the six primary cohorts. This yielded 75 KEGG metabolic subpathways for analysis.

Single-sample gene set enrichment analysis (ssGSEA) was performed separately within each cohort using the GSVA package and the cohort-specific processed expression matrix. ssGSEA scores were interpreted as inferred transcriptional pathway enrichment scores and not as direct measurements of metabolic flux. Because expression platforms and preprocessing differed across cohorts, raw ssGSEA scores were not directly compared across datasets. Instead, statistical models were fitted within each cohort and the resulting standardized effect estimates were combined by meta-analysis.

2.4 UC-versus-healthy/control KEGG pathway meta-analysis

For each of the 75 KEGG metabolic pathways, ssGSEA-derived pathway activity was compared between UC and healthy/control mucosa within each cohort. In GSE11223, the primary UC-versus-healthy/control model included disease status and colonic region as fixed effects and patient identity as a random intercept to account for repeated biopsies from the same individual. Terminal ileal samples were excluded from this analysis. In GSE206171, paired sigmoid biopsies were averaged to participant-level profiles before pathway scoring and modeling. Standard linear models were used for GSE38713, GSE206285, GSE53306, and GSE59071.

For each pathway and cohort, the UC-versus-healthy/control coefficient was expressed as a standardized effect size by scaling the UC-minus-healthy/control model coefficient to the residual standard deviation of

the corresponding model. Negative standardized effects indicated lower inferred pathway activity in UC, whereas positive standardized effects indicated higher inferred pathway activity in UC.
Cohort-specific standardized pathway effects and standard errors were combined across the six cohorts using random-effects meta-analysis with restricted maximum likelihood estimation. Pathways were included in the primary pooled analysis only when an estimable effect was available across all six cohorts. Meta-analysis P values were adjusted across the 75 KEGG metabolic pathways using the Benjamini-Hochberg procedure. Pathways with meta-analysis FDR < 0.05 were considered significantly altered in UC.
For visualization, the signed KEGG pathway landscape displayed the pooled standardized UC-versus-healthy/control effect on the x-axis and the negative $\log_{10}$-transformed meta-analysis FDR on the y-axis. The heatmap panel displayed cohort-specific standardized UC-minus-healthy/control effects for selected pathways. The main heatmap included the ten most significant FDR-significant pathways with lower activity in UC and all FDR-significant pathways with higher activity in UC. Heatmap asterisks indicated cohort-specific Benjamini-Hochberg FDR < 0.05, while text color was used only for readability.

2.5 Cross-cohort meta-analysis of SLC26A3/DRA expression

To determine whether DRA expression differed between healthy/control and UC mucosa, SLC26A3 expression was analyzed independently within each of the six primary cohorts using native processed expression values. Cohort-specific expression values were not merged across datasets because of differences in transcriptomic platform and preprocessing.
For the final DRA expression analysis, GSE11223 was restricted to the locked descending-colon subset containing 34 UC and 23 healthy/control samples. This was done to match the tissue region used in the companion DRA-focused transcriptomic validation. If repeated patient sampling was present in the GSE11223 descending-colon subset, DRA expression was modeled using a patient random intercept; otherwise, a standard linear model was used. The model compared SLC26A3 expression between UC and healthy/control samples. For GSE206171, participant-level averaged profiles were used. Standard linear models were used for the remaining cohorts.
For each cohort, the UC-minus-healthy/control DRA effect was standardized by the residual standard deviation of the corresponding model. Negative standardized effects indicated lower DRA expression in UC. Cohort-specific standardized effects and standard errors were combined using random-effects meta-analysis with restricted maximum likelihood estimation. Cohort-level P values were adjusted across the six DRA comparisons using the Benjamini-Hochberg procedure. The pooled standardized effect, 95% confidence interval, and meta-analysis P value were reported to summarize the overall direction and magnitude of DRA expression change in UC.
Cohort-level DRA expression distributions were visualized using native processed expression values plotted separately within each cohort. The accompanying forest plot displayed standardized cohort-level and pooled effects rather than pooled raw expression values. The final DRA figure panels were generated using the descending-colon GSE11223 analysis and the original locked definitions for the other five cohorts.

2.6 Robustness analyses for DRA expression meta-analysis

Sensitivity analyses were performed to evaluate whether the pooled reduction in DRA expression was driven by group-size imbalance or by a single influential cohort. These analyses used the final six-cohort DRA expression model, with GSE11223 restricted to descending colon and the other five cohorts retained under their locked definitions.
First, leave-one-cohort-out meta-analysis was performed by repeating the random-effects meta-analysis after removing one cohort at a time. This tested whether the pooled DRA reduction depended on any single cohort.
Second, repeated balanced resampling was performed to address unequal UC and healthy/control sample sizes. In each iteration, the larger group within each cohort was downsampled without replacement to

match the smaller group. The cohort-level DRA model was then refitted, and the six-cohort random-effects meta-analysis was recomputed. This procedure was repeated 1,000 times to generate an empirical distribution of pooled standardized DRA effects. Persistence of a negative pooled effect across resamples was interpreted as evidence that unequal group sizes were unlikely to explain the overall reduction in DRA expression.

Third, study-level influence diagnostics were calculated for the final six-cohort random-effects DRA meta-analysis. These diagnostics included Cook's distance, DFFITS, hat values, DFBETAS, externally standardized residuals, and combined influential-case flags. These analyses were used to identify cohorts contributing most strongly to the pooled estimate and to determine whether the direction and significance of the DRA reduction remained stable despite cohort influence.

2.7 Within-UC DRA-associated KEGG pathway analysis

To determine whether metabolic pathway activity was associated with DRA expression within UC, analyses were restricted to UC samples from the same six primary human colonic cohorts. DRA expression was defined as the processed expression value of SLC26A3 within each cohort. Because transcriptomic platforms and expression scales differed among studies, SLC26A3 expression was standardized as a z-score within the UC samples of each cohort before statistical modeling.

The same 75 KEGG metabolic pathways used in the UC-versus-healthy/control analysis were scored independently within each cohort using ssGSEA. For the DRA-association analysis, pathway scores were also standardized as z-scores within the UC samples of each cohort. Thus, the regression coefficient represented the change in standardized pathway activity per one standard deviation higher DRA expression within UC.

For the final DRA-focused analysis, GSE11223 was restricted to descending-colon UC samples. In this subset, each pathway score was modeled as a function of standardized DRA expression. If repeated patient sampling was present, a patient random-intercept model was attempted; if the mixed model was not estimable, a standard linear model was used as fallback. For GSE206171, paired sigmoid biopsies were averaged to participant-level profiles before pathway scoring and regression analysis. Standard linear regression models were used for the remaining cohorts.

Positive DRA-association effects indicated higher inferred pathway activity in UC samples with higher DRA expression. Negative DRA-association effects indicated lower inferred pathway activity in UC samples with higher DRA expression, or conversely higher pathway activity in the lower-DRA UC state. Cohort-specific regression coefficients and standard errors were combined using random-effects meta-analysis with restricted maximum likelihood estimation. Benjamini-Hochberg correction was applied across all 75 KEGG metabolic pathways, and pathways with meta-analysis FDR < 0.05 were considered significantly associated with DRA expression levels within UC.

2.8 Comparison of UC-associated and DRA-associated metabolic pathways

To determine whether DRA-associated pathways represented disease-associated metabolic programs, results from the primary UC-versus-healthy/control KEGG pathway meta-analysis were merged with results from the final within-UC DRA-associated pathway meta-analysis. This comparison included all pathways significant in the primary UC-versus-healthy/control analysis at FDR < 0.05, rather than only the pathways displayed in the main figure.

The two effect estimates were treated as independently derived standardized effects. The UC-versus-healthy/control pathway effect came from the primary Step 2A disease analysis, in which GSE11223 used all eligible colonic regions with region and patient-aware modeling. The within-UC DRA association came from the final DRA-focused analysis, in which GSE11223 was restricted to descending-colon UC samples.

Concordance was assessed by comparing the direction of pooled standardized effects between the two analyses. Pathways showing lower activity in UC together with positive DRA associations were interpreted

as disease-suppressed metabolic programs that were relatively preserved in UC samples with higher DRA expression. Pathways showing higher activity in UC together with negative DRA associations were interpreted as programs enriched in the low-DRA UC state. This comparison was used as a directional association audit and not as a causal or joint statistical test.
For visualization, representative DRA-associated pathways were displayed as a cross-cohort heatmap showing cohort-specific DRA associations, pooled DRA-association effects, and the corresponding pooled UC-versus-healthy/control disease effects. A separate supplementary comparison displayed all UC-significant pathways with their corresponding within-UC DRA associations.

2.9 Animal studies and mouse sample collection
All mouse studies were approved by the Institutional Animal Care and Use Committees of the Jesse Brown Veterans Affairs Medical Center and the University of Illinois Chicago. C57BL/6 WT and Slc26a3-deficient DRA KO mice aged 8-10 weeks were housed under specific pathogen-free conditions on a 12 h light/12 h dark cycle with ad libitum access to chow and water. To standardize hydration and mitigate dehydration risk in DRA KO animals, all mice, including WT controls, received an oral electrolyte solution throughout the study as previously described.
Mice of both sexes were used. Colonic mucosal RNA-seq was performed using WT and DRA KO mice with n = 3 biological replicates per group. Untargeted metabolomics of colonic mucosa, feces, and serum was performed using an independent WT and DRA KO cohort with n = 5 biological replicates per group. Targeted bile acid profiling and 16S rRNA-based microbial profiling were performed on the corresponding serum and fecal sample sets, as indicated for each analysis.
Mice were euthanized by CO_2 asphyxiation followed by cervical dislocation. After euthanasia, the colon was excised and colonic mucosa was collected by scraping, flash-frozen in liquid nitrogen, and stored at -80°C until analysis. Fresh fecal pellets were collected, snap-frozen, and stored at -80°C. Serum was isolated from whole blood collected by cardiac puncture after clotting for 30 min at room temperature and centrifugation at 2,000 × g for 10 min at 4°C. Serum aliquots were stored at -80°C until analysis.

2.10 Colonic mucosal RNA extraction, library preparation, and sequencing
Total RNA was extracted from colonic mucosa using the RNeasy Plus Micro Kit. Total RNA was treated with DNase I to remove residual genomic DNA, quantified using Qubit, and assessed using an Agilent 2100 Bioanalyzer. Samples with RNA integrity number (RIN) ≥ 7 were advanced to library preparation.
Directional, strand-specific mRNA libraries were prepared by the Northwestern University core facility from total RNA using the TruSeq Stranded mRNA kit according to the manufacturer's instructions. Briefly, poly(A)-selected RNA was chemically fragmented to approximately 200 bp, followed by first-strand cDNA synthesis with random hexamers and second-strand synthesis using RNase H and DNA Polymerase I. After magnetic-bead cleanup, end repair, A-tailing, and ligation of barcoded Illumina adapters, libraries were PCR-enriched. Library size, quality, and concentration were verified by Bioanalyzer and qPCR.

Equimolar library pools were sequenced on an Illumina HiSeq 4000 platform to generate single-end 50-bp reads. Library strandedness corresponded to first-strand, reverse orientation. Adapter sequences and low-quality bases were trimmed. Cleaned reads were aligned to the mouse reference genome GRCm39 using Ensembl release 110 annotation and STAR v2.7. Strand-aware gene-level raw count matrices were generated from the alignments and used for downstream analysis.

2.11 Mouse RNA-seq differential-expression analysis

Raw gene-level count data from colonic mucosa of WT and DRA KO mice were analyzed in R. The analysis included three WT and three DRA KO biological replicates. Differential expression was assessed using DESeq2 with genotype as the experimental factor, comparing DRA KO versus WT colonic mucosa.

Differential-expression testing was performed using the DESeq2 Wald test. For each gene, DESeq2 estimated the $\log_2$ fold change, standard error, Wald statistic, nominal P value, and Benjamini-Hochberg FDR. Genes with Benjamini–Hochberg FDR < 0.05 were considered significantly differentially expressed. Positive $\log_2$ fold-change values indicated higher expression in DRA KO colonic mucosa, whereas negative values indicated higher expression in WT mucosa or reduced expression in DRA KO. These directional assignments were used throughout the analysis as KO-upregulated and WT-high/KO-downregulated genes.

For downstream visualization and correlation analyses, normalized expression values were generated using the DESeq2 variance-stabilizing transformation. Variance-stabilized expression values were used for metabolism-focused PCA, prioritized-gene heatmaps, pathway expression-score calculation, gene-gene correlation analysis, and pathway-correlation analysis.

2.12 KEGG metabolism-focused transcriptomic analysis

To focus the transcriptomic analysis on metabolism-associated genes, mouse genes with testable differential-expression statistics were mapped to KEGG metabolism pathways. The KEGG metabolism gene universe was defined as the set of testable genes assigned to the selected KEGG metabolism pathway collection. This metabolism-focused gene universe contained 1,515 testable genes.

Genes were mapped to KEGG metabolic pathways using gene-to-pathway membership annotations. Because individual genes can be assigned to more than one KEGG pathway, pathway-level gene counts were treated as annotation-level summaries and were not considered mutually exclusive. Therefore, a single gene could contribute to more than one pathway-level summary if it was annotated to multiple KEGG metabolism pathways. For graphical display, KEGG pathways were grouped into broad metabolism categories, including amino acid metabolism, carbohydrate metabolism, cofactors and vitamins metabolism, energy metabolism, glycan metabolism, lipid and isoprenoid metabolism, nucleotide metabolism, and xenobiotic or drug metabolism.

Gene set enrichment analysis (GSEA) was performed to identify KEGG metabolism pathways showing coordinated transcriptomic differences between DRA KO and WT colonic mucosa. Genes were ranked using the DESeq2 Wald statistic from the DRA KO versus WT comparison. This statistic-ranked list retained the direction and strength of gene-level differences for pathway-level testing. Pathways with Benjamini-Hochberg FDR < 0.05 were considered significantly enriched. These GSEA results were used to classify KEGG metabolism pathways as significant or non-significant in the pathway summary. This approach allowed detection of coordinated pathway-level shifts even when only a subset of member genes reached individual differential-expression significance.

2.13 Metabolism-focused PCA and gene-loading analysis

To evaluate the structure of metabolism-associated gene-expression variation, PCA was performed using variance-stabilized expression values restricted to KEGG metabolism genes. Before PCA, expression values were standardized to gene-wise z-scores across the six samples so that each gene contributed according to its relative expression pattern rather than its absolute expression level.

PCA was performed on the metabolism-focused expression matrix, and gene loadings for principal components 1 and 2 were extracted. The loading plot was used to visualize how individual metabolic genes contributed to the dominant axes of metabolic transcriptional variation. The PC1 orientation was displayed for interpretability, with one direction corresponding to WT-associated gene-loading patterns and the opposite direction corresponding to DRA KO-associated gene-loading patterns.

Each point in the PCA loading plot represented one KEGG metabolism-associated gene. Non-significant metabolic genes were displayed as background points, whereas DESeq2-significant genes were highlighted according to their direction of differential expression in DRA KO versus WT. Point colors indicated broad KEGG metabolism category. Genes with strong positive or negative PC1 loadings were interpreted as major contributors to the metabolism-focused WT-associated or DRA KO-associated transcriptional axis. Absolute PC1 loadings were also used as one component of the downstream gene-prioritization and pathway-contribution analyses.

2.14 Integrated prioritization of metabolic genes

FDR-significant metabolic DEGs were prioritized using complementary features that captured statistical strength, effect size, contribution to the main metabolism-focused PCA axis, and gene–gene correlation connectivity. Specifically, the prioritization considered DESeq2 FDR, absolute $\log_2$ fold change for DRA KO versus WT, absolute PC1 loading from the metabolism-focused PCA, and total retained gene-gene correlation degree.

This prioritization was used to select high-priority WT-associated and DRA KO-associated metabolic genes for visualization. The selected genes were displayed using a paired heatmap and lollipop plot. The heatmap showed z-scored normalized expression across individual WT and DRA KO samples. The lollipop plot showed $\log_2$ fold change for DRA KO versus WT, DESeq2 FDR values, broad KEGG metabolism category, and gene-gene correlation connectivity. These genes were interpreted as prioritized metabolic candidates rather than functionally validated driver genes.

2.15 Gene-gene correlation connectivity

To estimate the connectivity of prioritized metabolic genes within the FDR-significant metabolic DEG set, pairwise gene-gene correlations were calculated using normalized expression values across the six samples. Spearman correlation was used to quantify monotonic relationships between each prioritized gene and other FDR-significant metabolic DEGs. For each gene pair, a Spearman rho and nominal P value were calculated, and P values were adjusted using the Benjamini-Hochberg procedure.

A gene-gene correlation was retained when it met both of the following criteria: absolute Spearman rho $\geq$ 0.80 and FDR $<$ 0.10. Retained positive and negative correlations were summarized separately and together. For each prioritized gene, total retained correlation degree was calculated as the number of retained positive and negative correlations formed with other significant metabolic DEGs. This total retained degree was used as the point-size variable in the lollipop plot.

2.16 Pathway-level DEG burden and PC1 contribution analysis

To summarize differential expression at the pathway level, FDR-significant metabolic DEGs were assigned to KEGG metabolism pathways according to pathway membership. Within each pathway, significant genes were separated by direction of differential expression. Genes with positive $\log_2$ fold-change values were counted as KO-upregulated genes, whereas genes with negative $\log_2$ fold-change values were counted as WT-high/KO-downregulated genes.

For each KEGG metabolism pathway, the KO-upregulated DEG burden and WT-high/KO-downregulated DEG burden were calculated as the number of significant metabolic genes assigned to that pathway in each direction. These values were visualized as mirrored bars to show directional pathway-level DEG burden. Because KEGG pathway membership is overlapping, genes annotated to multiple pathways contributed to each corresponding pathway-level count. Therefore, pathway DEG burdens were interpreted as annotation-level summaries rather than independent gene counts.

To complement the pathway DEG burden analysis, pathway-level contribution to the metabolism-focused PCA axis was calculated using gene PC1 loadings. For each KEGG metabolism pathway, the absolute PC1 loadings of pathway member genes were summed. This value represented the total contribution of

pathway member genes to PC1, independent of the direction of loading. The summed absolute PC1 contribution was displayed alongside directional DEG burden to highlight pathways contributing to the metabolism-focused transcriptional structure through either a high number of significant DEGs, strong PC1 loading contribution among pathway member genes, or both.

2.17 Pathway expression-score calculation and pathway-correlation analysis

To examine coordinated pathway-level expression patterns, pathway expression scores were calculated for each KEGG metabolism pathway in each sample. First, variance-stabilized expression values for metabolism-associated genes were standardized to gene-wise z-scores across samples. For each pathway, the pathway expression score for each sample was calculated as the mean z-scored expression value of all genes assigned to that pathway.

Pairwise correlations were then calculated among KEGG metabolism pathway expression scores across the six samples. Spearman correlation was used to quantify monotonic relationships between pathway-level expression patterns. For each pathway pair, Spearman rho and nominal P values were calculated. P values were adjusted across all pathway pairs using the Benjamini-Hochberg procedure.

Because KEGG pathways can share member genes, gene-overlap filtering was applied to reduce correlations driven primarily by overlapping pathway annotations. For each pathway pair, Jaccard gene overlap was calculated as the number of shared genes divided by the total number of unique genes assigned to either pathway. A pathway correlation was retained only if it satisfied all of the following criteria: absolute Spearman rho ≥ 0.80, FDR < 0.10, and Jaccard gene overlap < 0.25. Retained positive correlations represented pathway pairs with similar expression-score patterns across samples, whereas retained negative correlations represented pathway pairs with opposing expression-score patterns. In pathway-correlation heatmaps, colored cells indicated retained pathway correlations, while gray cells indicated non-retained pathway pairs included for local module context. Retained correlations meeting the more stringent FDR < 0.05 threshold were marked with an asterisk.

2.18 Hierarchy-guided local pathway-correlation modules and pathway hubs

To visualize pathway-level organization, a signed pathway-correlation matrix was generated from pairwise Spearman correlations among KEGG metabolism pathway scores. Pathways were ordered using hierarchical clustering of the signed correlation matrix with average linkage and a correlation-derived distance metric.

Representative local pathway-correlation modules were selected from the hierarchy-guided pathway-correlation structure to display biologically coherent pathway neighborhoods. Four local modules were shown: energy-redox/central carbon, amino acid-lipid/bile acid, carbohydrate-CYP/lipid, and cofactor-sugar/glycolipid. These modules were selected to summarize major coordinated metabolic structures observed in the pathway-correlation network. Non-retained pathway pairs within each selected local module were retained as gray cells to preserve local matrix structure and to avoid displaying only isolated retained correlations. The displayed local modules were selected to include the major pathway-correlation hubs represented in the global hub analysis.

A pathway hub was defined by retained pathway-correlation degree. For each KEGG metabolism pathway, retained degree was calculated as the number of distinct pathways with which it formed a retained correlation, using the retained-edge criteria of absolute Spearman rho ≥ 0.80, FDR < 0.10, and Jaccard gene overlap < 0.25. Positive and negative retained correlations were summarized separately, while total retained correlation degree was used for hub ranking.

The top 10 pathway-correlation hubs were ranked according to total retained correlation degree and displayed as a bar plot. These hubs represented pathways with the greatest number of retained pathway-level associations across the KEGG metabolism network. Pathway names were abbreviated for graphical

display using a fixed abbreviation dictionary. Full KEGG pathway names and corresponding abbreviations were provided in the supplementary annotation table.

2.19 Untargeted LC-MS metabolomics of colon, feces, and serum

Untargeted metabolomics of colonic mucosa, feces, and serum was performed at the Southeast Center for Integrated Metabolomics (SECIM), University of Florida. Samples were normalized before extraction according to the SECIM workflow, including total protein-based normalization where applicable. Analyses were performed on a Thermo Q Exactive Orbitrap mass spectrometer coupled to a Dionex UHPLC system and autosampler. Samples were analyzed in separate injections under positive- and negative-ion heated electrospray ionization modes. Chromatographic separation was performed on an ACE 18-PFP column (100 × 2.1 mm, 2 μm) using 0.1% formic acid in water as mobile phase A and acetonitrile as mobile phase B. The flow rate was 350 μL/min, and the column temperature was maintained at 25°C. Injection volumes were 4 μL for negative-ion mode and 2 μL for positive-ion mode. Data were acquired at a resolution of 35,000 at m/z 200.

Raw LC-MS data were processed by SECIM using MZmine for feature detection, deisotoping, alignment, and preliminary metabolite annotation. Adducts and complexes were identified and removed from the downstream curated feature tables where appropriate. Mass and retention-time data were searched against internal metabolite libraries, and known metabolites were mapped to KEGG and HMDB identifiers when available. SECIM provided processed feature-intensity tables for each compartment and ionization mode, together with metabolite labels, retention times, mass-to-charge ratios, adduct assignments, MSI annotation levels, and available KEGG and HMDB identifiers. Because individual LC-MS features can represent ion forms, adducts, or related signals rather than unique metabolites, original feature identifiers and annotation metadata were retained throughout downstream curation.

Quality-control processing included blank feature filtering using interquartile-range-based filtering and missing-value imputation using k-nearest neighbor imputation, as implemented in MetaboAnalystR where applicable. These steps were applied before downstream statistical and pathway analyses.

2.20 MetaboAnalyst-based statistical and multivariate analysis

SECIM-generated processed feature-intensity tables were used as input for downstream metabolomics analysis in MetaboAnalyst. Positive- and negative-ion-mode datasets were analyzed separately for colonic mucosa and feces. For serum untargeted metabolomics, the main analysis focused on the negative-ion-mode dataset, with positive-ion-mode serum results included as supplementary analysis where applicable. Before downstream analysis, curated feature tables were normalized using probabilistic quotient normalization with a pooled WT reference sample, followed by $\log_{10}$ transformation and Pareto scaling. These preprocessing steps were applied to reduce unwanted intensity variation, stabilize variance, and balance the contribution of high- and low-abundance features.

Principal component analysis, PERMANOVA, partial least-squares discriminant analysis, variable importance in projection scoring, univariate testing, fold-change analysis, heatmap generation, and volcano-plot generation were performed in MetaboAnalyst. PLS-DA models were evaluated using 5-fold cross-validation to estimate model performance, including R^2 and Q^2 values, and permutation testing with 1,000 permutations was used to assess overfitting. The PLS-DA component with the highest Q^2 value was prioritized for interpretation when selecting VIP-ranked features.

Differential metabolite features were identified using pairwise t-tests with Benjamini-Hochberg FDR correction. Features were considered significantly altered when the FDR-adjusted P value was < 0.05 and the KO-to-WT fold change was ≥ 1.30 or ≤ 0.769. VIP scores were used for feature prioritization, with VIP $\geq$ 1.5 used as the prioritization threshold. PLS-DA and VIP results were interpreted alongside univariate statistics, annotation confidence, and pathway context rather than as stand-alone confirmatory evidence.

For downstream curation, MetaboAnalyst statistical outputs were linked back to the corresponding SECIM annotation tables for each compartment and ionization mode using feature labels and retention-time information. Original LC-MS feature labels, ionization mode, adduct assignments, MSI annotation levels, and feature-level statistical results were retained. Features representing related ion forms across positive- and negative-ion modes were grouped under provisional neutral metabolite labels for pathway preparation while preserving their original feature-level information.

2.21 Metabolite prioritization and MetaboAnalyst pathway topology analysis

Four complementary metabolite-prioritization layers were generated independently for colon, feces, and serum. Layer 1 included all significant annotated metabolites. Layer 2 included significant metabolites annotated at MSI level 1. Layer 3 included MSI level 1 metabolites with a maximum PLS-DA VIP score $\geq$ 1.5 across reported components. Layer 4 included MSI level 3 metabolites with a maximum VIP score $\geq$ 1.5. These layers partially overlapped and were interpreted as complementary prioritization strategies rather than independent metabolite datasets. Metabolites remained in their assigned layer regardless of whether a KEGG identifier was available; KEGG and HMDB identifiers were retained as annotation metadata and were not used as inclusion criteria for layer assignment.

Pathway topology analysis of untargeted metabolomics data was performed using the MetaboAnalyst web platform. Analyses were performed separately for each compartment and prioritization layer against the Mus musculus KEGG pathway library. Hypergeometric over-representation analysis was used for pathway enrichment, and relative-betweenness centrality was used for pathway topology assessment. Pathways with FDR < 0.05 were considered statistically significant.

MetaboAnalyst pathway FDR values refer to pathway enrichment results from the hypergeometric over-representation test; VIP thresholds were used only to define prioritization layers and were not treated as pathway-level significance tests.

For visualization of cross-layer consistency, pathways with FDR < 0.10 in at least one layer within a compartment were displayed in tissue-specific dot heatmaps. Dot color represented $-\log_{10}$(FDR), dot size represented pathway impact, filled symbols indicated FDR < 0.05, and open symbols indicated suggestive enrichment with $0.05 \leq$ FDR < 0.10. Layers 1 and 2 were interpreted as significance-led evidence, whereas Layers 3 and 4 were interpreted as VIP-prioritized supportive analyses. Because Layer 4 was derived from MSI level 3 annotations, its pathway results were considered exploratory.

2.22 Transcriptome-metabolome pathway-level convergence analysis

Because colonic transcriptomic and metabolomic datasets were generated from independent mouse cohorts, integration was performed at the pathway level rather than by sample-wise gene-metabolite correlation. The goal was to determine whether metabolic pathways altered at the transcriptomic level in DRA KO colonic mucosa were also supported by metabolite-level changes in the colonic mucosal metabolome.

For the transcriptomic component, significant metabolism-associated DEGs were defined as genes assigned to the KEGG metabolism gene universe and differentially expressed between DRA KO and WT colonic mucosa at FDR < 0.05. For the metabolomic component, high-confidence metabolite evidence was prioritized using the MSI level 1 significance layer, consisting of metabolites that were significantly altered by univariate analysis and annotated at MSI level 1.

Genes and metabolites were mapped to KEGG metabolic pathways, and pathway-level overlap was summarized by comparing the number of significant transcriptomic genes and matched metabolite features assigned to each pathway. Shared pathways were defined as pathways supported by both significant metabolism-associated RNA-seq changes and significant high-confidence metabolite evidence. Representative shared pathways were visualized by mapping pathway-associated genes and metabolites onto KEGG pathway diagrams, with gene and metabolite nodes colored according to $\log_2$ fold change in

DRA KO versus WT. In integrated pathway diagrams, gene and metabolite nodes were displayed using distinct shapes and colored according to $\log_2$ fold change in DRA KO versus WT.
This analysis was interpreted as pathway-level convergence between independent transcriptomic and metabolomic datasets. It was not interpreted as direct evidence of sample-wise gene-metabolite regulation, causal gene-metabolite interaction, or reaction-based gene-metabolite connectivity.

2.23 Targeted bile acid profiling and bile acid class analysis
Targeted bile acid profiling was performed in serum and fecal samples from WT and DRA KO mice by LC-MS/MS at the McGuire Research Institute Mass Spectrometry Facility. Samples were extracted using an acetonitrile:methanol-based preparation with isotope-labeled internal standards and quantified using batch-specific 9-point calibration curves.
Chromatographic separation was performed on a Thermo Scientific Hypersil Gold column (100 × 2.1 mm, 1.9 µm). Bile acids were analyzed in negative ESI mode on a Shimadzu LCMS-8060 CL triple quadrupole instrument. Delivered concentration matrices were reported as nmol/L for serum and nmol/g for feces; therefore, serum and fecal bile acid datasets were analyzed separately.
Individual bile acids were annotated as primary, secondary, or other and further classified according to conjugation state and sulfation status. Summary bile acid metrics were calculated separately for serum and feces, including total bile acids, secondary bile acid fraction, DCA/CA ratio, conjugated bile acid fraction, unconjugated bile acid fraction, and sulfated bile acid fraction. Feature-level and class-level WT-versus-DRA KO comparisons were performed separately within serum and feces using Wilcoxon rank-sum tests. Benjamini–Hochberg-adjusted FDR values were calculated within each compartment for multiple-testing assessment. For Figure 5e–f, asterisks denote nominal, unadjusted P values: $^*P < 0.05$ and $^{**}P < 0.01$. Heatmaps display row-scaled z scores calculated separately within each compartment.

2.24 MetOrigin source attribution and co-metabolism analysis
Differentially abundant metabolite features from colonic mucosa, feces, and serum were analyzed using MetOrigin 2.0 to infer possible metabolite source attribution. For each compartment, significant features from positive- and negative-ion modes were combined and deduplicated before submission to MetOrigin. MetOrigin was used to classify metabolites into host-associated, microbiota-associated, host-microbiota co-metabolism, food-associated, drug-associated, environment-associated, and unknown categories. Categories were not treated as mutually exclusive.
Pathway enrichment was performed within each compartment and origin class using MetOrigin. P values were adjusted using the Benjamini-Hochberg procedure within each compartment and origin group. For visualization, altered metabolite feature counts, source assignments, source-overlap diagrams, origin-specific pathway enrichment, and co-metabolism pathway summaries were generated. Alluvial plots were used to display links among selected co-metabolism pathways, implicated microbial enzymes, and associated genera. MetOrigin assignments were interpreted as computational inferences of possible source contribution and not as direct evidence of microbial production, host production, or host-microbial metabolic flux.

2.25 16S rRNA sequencing and genus-level microbial analysis
Fecal microbial community profiling was used to compare genotype-associated microbial shifts with the MetOrigin-derived co-metabolic framework. Samples were processed by the ZymoBIOMICS Targeted Sequencing Service using bacterial 16S rRNA gene amplicon sequencing with the Quick-16S NGS Library Prep Kit, targeting the V3-V4 region of the 16S rRNA gene in most cases. Libraries were sequenced on an Illumina MiSeq using a v3 600-cycle reagent kit.
Amplicon sequence variants were inferred using DADA2, and taxonomy was assigned using Uclust in QIIME v1.9.1 against the Zymo Research Database. For the present study, WT and DRA KO fecal samples

were analyzed with n = 5 per group. Genus labels were extracted from the taxonomic abundance table, and abundances mapping to the same genus were summed to generate genus-level relative abundance values. Analyses were restricted to genera represented in the MetOrigin co-metabolism pathway-enzyme-genus framework. WT-versus-DRA KO comparisons were performed using Wilcoxon rank-sum tests. These analyses were used to determine whether genera implicated by MetOrigin were detectable and directionally altered in the microbiome data. For genus-level plots, asterisks denoted raw Wilcoxon rank-sum test significance. These genus-level comparisons were interpreted descriptively because analyses were restricted to genera represented in the MetOrigin co-metabolism pathway-enzyme-genus framework.

2.26 Statistical analysis and visualization

All statistical analyses were performed using R version 4.5.2, unless otherwise specified. Mouse RNA-seq differential-expression analysis was performed using DESeq2. Single-sample gene set enrichment analysis was performed using GSVA. KEGG pathway definitions and gene annotations were generated using KEGGREST, AnnotationDbi, org.Hs.eg.db, and org.Mm.eg.db where applicable. Mixed-effects models were fitted using nlme, standard linear models using the stats package, and random-effects meta-analysis using metafor with restricted maximum likelihood estimation. Data manipulation and visualization were performed using tidyverse-based workflows, ggplot2, ggrepel, and ComplexHeatmap where applicable.

Untargeted metabolomics preprocessing, statistical analyses, and pathway topology analyses were performed using MetaboAnalyst and MetaboAnalystR where applicable. Source-attribution and host-microbiota co-metabolism analyses were performed using MetOrigin 2.0. For metabolomics, feature-level FDR, pathway-level FDR, and VIP prioritization were reported as separate evidence layers.

Targeted bile acid and genus-level microbiome comparisons were performed using Wilcoxon rank-sum tests unless otherwise indicated. Multiple-testing correction was performed using the Benjamini-Hochberg method. Unless otherwise specified, FDR < 0.05 was considered statistically significant. Figure-specific use of nominal P values is defined in the corresponding figure legends.

For mouse RNA-seq, significant DEGs were defined as Benjamini–Hochberg FDR < 0.05. For untargeted metabolomics, significantly altered features were defined as FDR < 0.05 with KO-to-WT fold change ≥ 1.30 or ≤ 0.769. For gene-gene and pathway-pathway correlation analyses, retained correlations required absolute Spearman rho ≥ 0.80 and FDR < 0.10; pathway correlations additionally required Jaccard gene overlap < 0.25. Retained pathway correlations with FDR < 0.05 were denoted with an asterisk in pathway-correlation heatmaps.

For heatmap visualization of standardized effects, green indicated positive effects or associations and pink indicated negative effects or associations, unless otherwise specified in the figure legend.

2.27 Data and resource availability

Raw RNA-seq FASTQ files are available in the NCBI Sequence Read Archive under BioProject PRJNA1345427. Untargeted LC-MS metabolomics data are available in GNPS/MassIVE under accession numbers MSV000099473 for positive mode and MSV000099476 for negative mode. This document provides detailed experimental protocols, analysis parameters, and study-specific workflow descriptions used for the transcriptomic, metabolomic, bile acid, MetOrigin, and 16S microbiome analyses. Derived summary tables generated from these analyses are included as accompanying repository files where applicable. Computational analyses were performed in R version 4.5.2, MetaboAnalyst, MetOrigin 2.0, and the analysis platforms specified above.
