# Supplementary material for: Multi-Compartment Metabolic Remodeling in DRA-Deficient Mice Overlaps with Ulcerative Colitis Metabolic Signatures
Source: Metabolites. 2026 Sep 18;16(9):690. doi: 10.3390/metabo16090690 (PMC13609582; doi:10.3390/metabo16090690)
Supplement: Supplementary file 1 [file metabolites-16-00690-s001.zip › Supplementary Figures/Supplementary Figure S2.pdf]

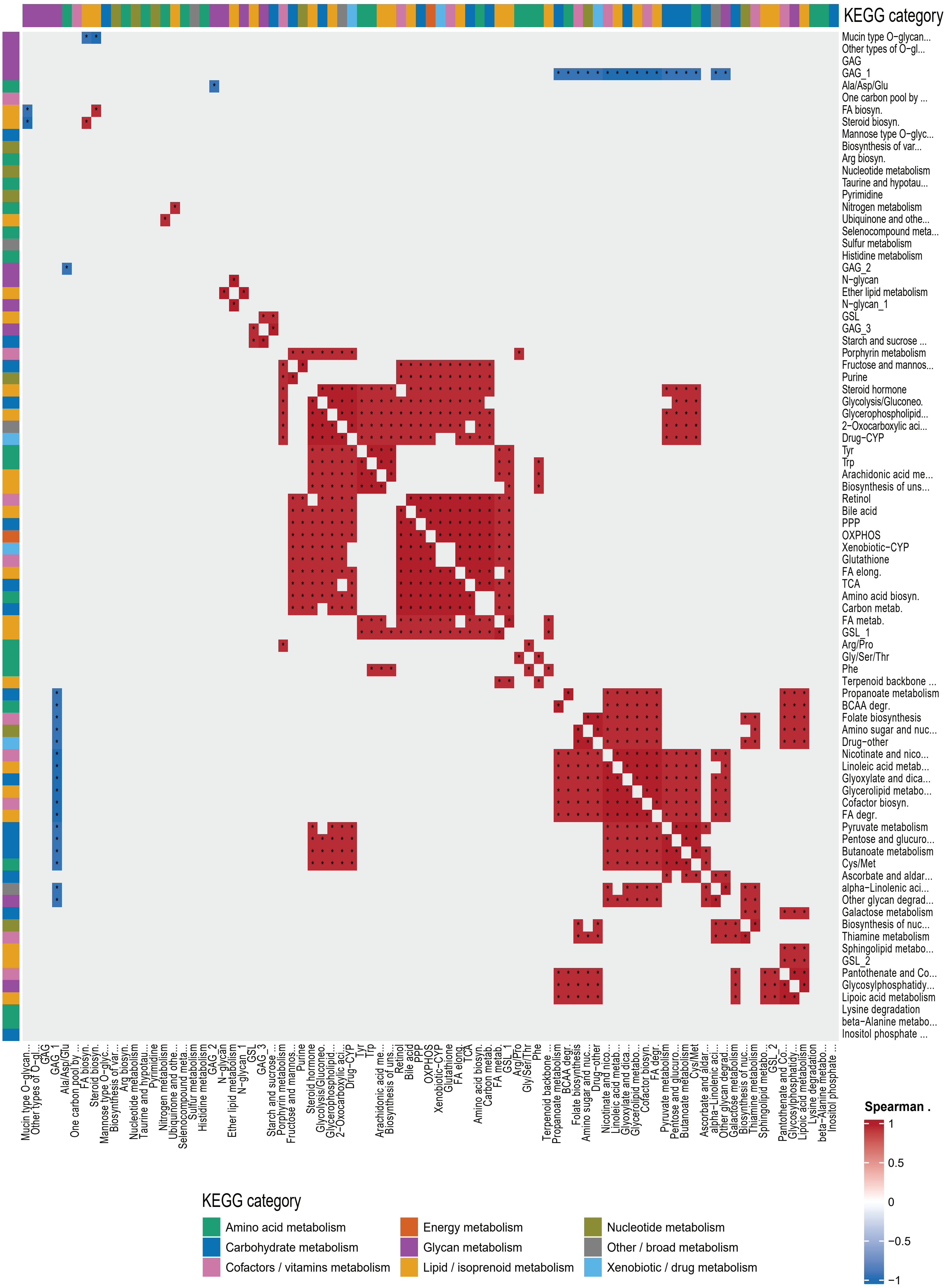

KEGG category
Mucin type O−glycan...
Other types of O−gl...
GAG
GAG_1
Ala/Asp/Glu
One carbon pool by ...
FA biosyn.
Steroid biosyn.
Mannose type O−glyc...
Biosynthesis of var...
Arg biosyn.
Nucleotide metabolism
Taurine and hypotau...
Pyrimidine
Nitrogen metabolism
Ubiquinone and othe...
Selenocompound meta...
Sulfur metabolism
Histidine metabolism
GAG_2
N−glycan
Ether lipid metabolism
N−glycan_1
GSL
GAG_3
Starch and sucrose ...
Porphyrin metabolism
Fructose and mannos...
Purine
Steroid hormone
Glycolysis/Gluconeo.
Glycerophospholipid...
2−Oxocarboxylic aci...
Drug−CYP
Tyr
Trp
Arachidonic acid me...
Biosynthesis of uns...
Retinol
Bile acid
PPP
OXPHOS
Xenobiotic−CYP
Glutathione
FA elong.
TCA
Amino acid biosyn.
Carbon metab.
FA metab.
GSL_1
Arg/Pro
Gly/Ser/Thr
Phe
Terpenoid backbone ...
Propanoate metabolism
BCAA degr.
Folate biosynthesis
Amino sugar and nuc...
Drug−other
Nicotinate and nico...
Linoleic acid metab...
Glyoxylate and dica...
Glycerolipid metabo...
Cofactor biosyn.
FA degr.
Pyruvate metabolism
Pentose and glucuro...
Butanoate metabolism
Cys/Met
Ascorbate and aldar...
alpha−Linolenic aci...
Other glycan degrad...
Galactose metabolism
Biosynthesis of nuc...
Thiamine metabolism
Sphingolipid metabo...
GSL_2
Pantothenate and Co...
Glycosylphosphatidy...
Lipoic acid metabolism
Lysine degradation
beta−Alanine metabo...
Inositol phosphate ...
Spearman .
1
0.5
0
−0.5
−1
KEGG category
Amino acid metabolism
Carbohydrate metabolism
Cofactors / vitamins metabolism
Energy metabolism
Glycan metabolism
Lipid / isoprenoid metabolism
Nucleotide metabolism
Other / broad metabolism
Xenobiotic / drug metabolism
