# Supplementary material for: Multi-Compartment Metabolic Remodeling in DRA-Deficient Mice Overlaps with Ulcerative Colitis Metabolic Signatures
Source: Metabolites. 2026 Sep 18;16(9):690. doi: 10.3390/metabo16090690 (PMC13609582; doi:10.3390/metabo16090690)

SUPPLEMENTARY FIGURE S4

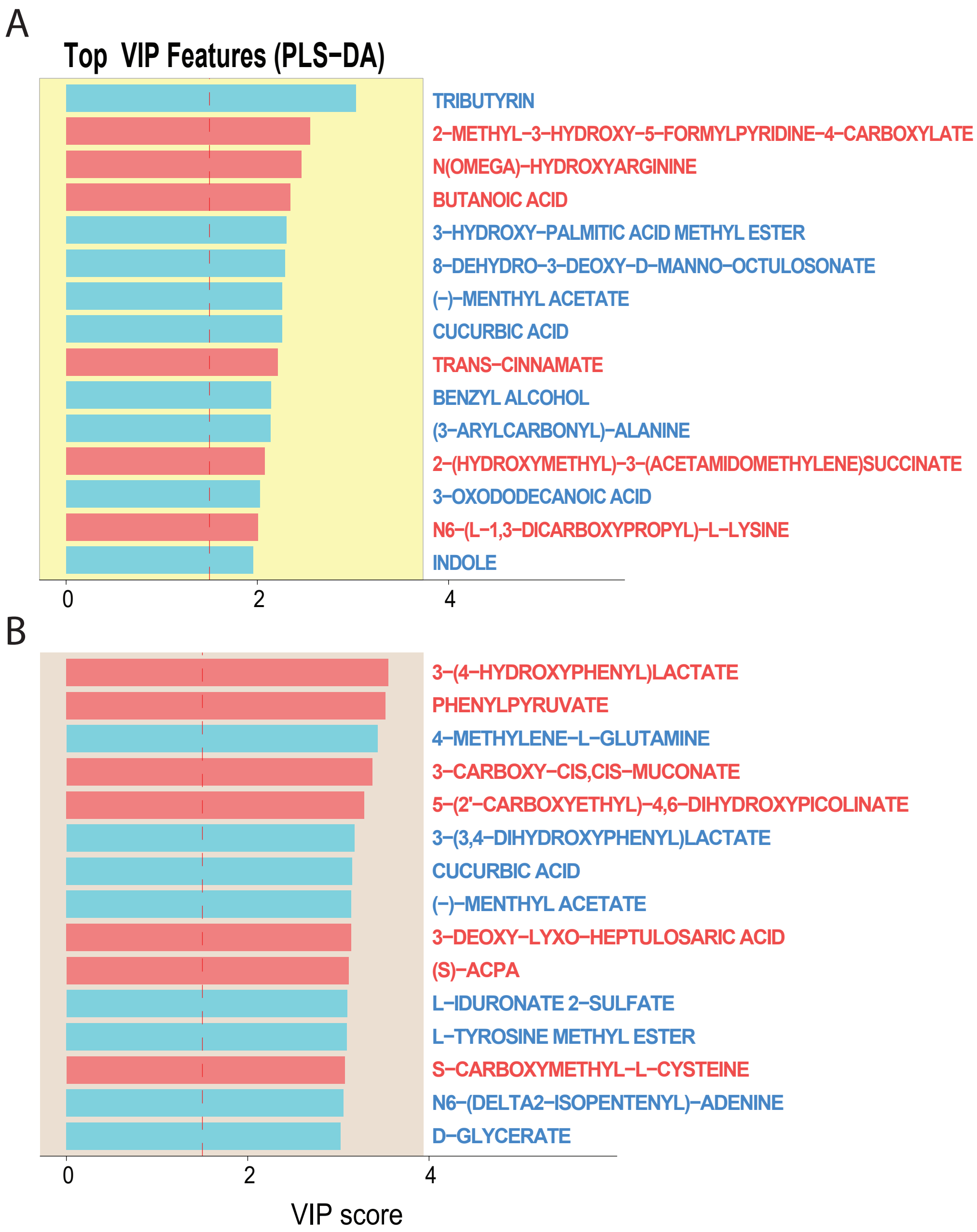

Supplement: Supplementary file 1 [file metabolites-16-00690-s001.zip › Supplementary Figures/Supplementary Figure S4.pdf]
