# Supplementary material for: Multi-Compartment Metabolic Remodeling in DRA-Deficient Mice Overlaps with Ulcerative Colitis Metabolic Signatures
Source: Metabolites. 2026 Sep 18;16(9):690. doi: 10.3390/metabo16090690 (PMC13609582; doi:10.3390/metabo16090690)
Supplement: Supplementary file 1 [file metabolites-16-00690-s001.zip › Supplementary Figures/Supplementary Figures and Figure legends.pdf]

Supplementary Fig. S1

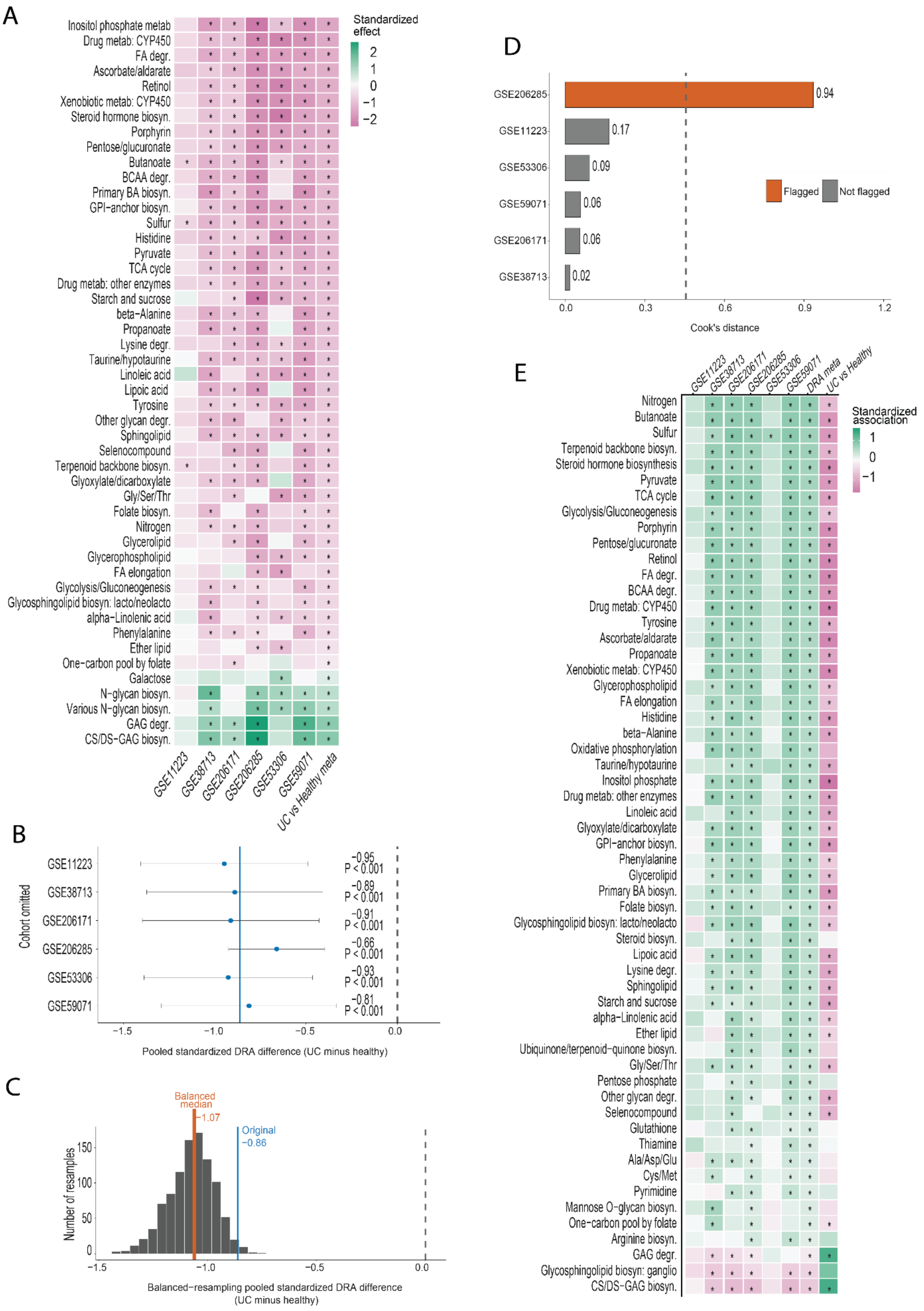

Supplementary Fig. S1 Expanded cross-cohort pathway and DRA meta-analysis. (a) Full heatmap of all KEGG metabolic pathways significantly altered in UC relative to healthy/control mucosa after pooled meta-analysis FDR correction. Rows represent the 48 FDR-significant pathways identified from the 75 KEGG metabolic pathways tested. The first six columns show cohort-specific standardized UC minus healthy/control effects for GSE11223, GSE38713, GSE206171, GSE206285, GSE53306, and GSE59071. The final column shows the random-effects pooled UC versus healthy/control pathway effect. Pink indicates lower inferred pathway activity in UC, whereas green indicates higher inferred pathway activity in UC. Asterisks indicate FDR < 0.05 for the corresponding cohort-level or pooled effect. **(b)** Leave-one-out sensitivity analysis for the DRA/SLC26A3 expression meta-analysis. Each row shows the pooled standardized DRA expression difference after omitting one cohort at a time. Negative values indicate lower DRA expression in UC compared with healthy/control mucosa. The dashed vertical line indicates no difference. **(c)** Balanced-resampling analysis of the pooled standardized DRA expression difference. The histogram shows the distribution of pooled effects across balanced resampling iterations. The blue vertical line indicates the original pooled estimate, and the orange vertical line indicates the balanced-resampling median. **(d)** Influence analysis showing Cook's distance for each cohort in the DRA expression meta-analysis. The dashed vertical line indicates the influence threshold used for flagging. GSE206285 showed the greatest influence, but the pooled DRA effect remained negative in sensitivity analyses. **(e)** Full heatmap of KEGG pathways significantly associated with continuous DRA expression within UC samples. Rows represent the 57 pathways with pooled DRA-association FDR < 0.05. The first six columns show cohort-specific standardized associations between pathway activity and DRA expression among UC samples only. The DRA meta column shows the random-effects pooled association between continuous DRA expression and pathway activity across the six UC cohorts. The UC vs Healthy column shows the independently derived random-effects pooled UC versus healthy/control pathway effect for comparison. Green indicates positive DRA-pathway associations or higher pathway activity in UC, whereas pink indicates negative DRA-pathway associations or lower pathway activity in UC. Asterisks indicate FDR < 0.05. **Abbreviations:** UC, ulcerative colitis; DRA, down-regulated in adenoma; SLC26A3, solute carrier family 26 member 3; KEGG, Kyoto Encyclopedia of Genes and Genomes; FDR, false discovery rate; GAG, glycosaminoglycan; CS/DS, chondroitin sulfate/dermatan sulfate; GAG, glycosaminoglycan; FA, fatty acid; BA, bile acid; BCAA, branched-chain amino acid; TCA cycle, tricarboxylic acid cycle; GPI, glycosylphosphatidylinositol; CYP450, cytochrome P450; Gly/Ser/Thr, glycine/serine/threonine metabolism; Cys/Met, cysteine/methionine metabolism; Ala/Asp/Glu, alanine/aspartate/glutamate metabolism; Gluconeo., gluconeogenesis; biosyn., biosynthesis; degr., degradation; metab., metabolism; Drug metab., drug metabolism; DRA meta, random-effects pooled DRA-pathway association across UC samples.

Supplementary Fig. S2

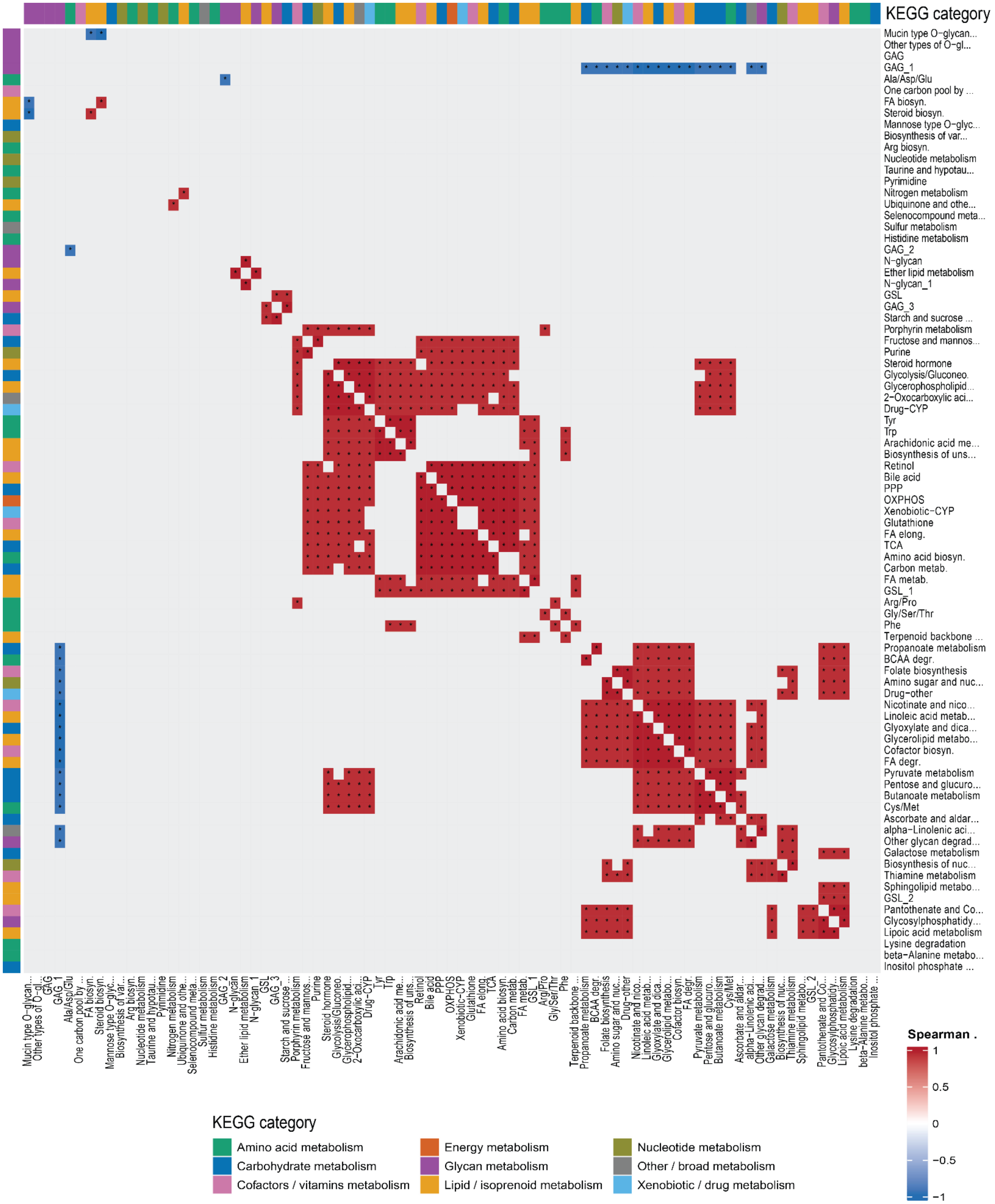

Supplementary Fig. S2 Full KEGG metabolism pathway-score correlation heatmap. Hierarchically clustered heatmap showing retained Spearman correlations among 83 KEGG metabolism pathway expression scores calculated from colonic RNA-seq data from WT and DRA KO mice. Pathway scores were derived from gene-wise standardized expression values for genes assigned to each KEGG metabolism pathway. Colored cells indicate retained pathway-pathway correlations meeting the criteria $|rho| \geq 0.80$, FDR < 0.10, and Jaccard gene-set overlap < 0.25. Red indicates positive correlations and blue indicates negative correlations. Asterisks indicate correlations with FDR < 0.05. Top and side annotation bars indicate KEGG metabolic categories. **Abbreviations:** WT, wild-type; KO, knockout; DRA, down-regulated in adenoma; KEGG, Kyoto Encyclopedia of Genes and Genomes; FDR, false discovery rate; rho, Spearman correlation coefficient; GAG, glycosaminoglycan; GSL, glycosphingolipid; FA, fatty acid; PPP, pentose phosphate pathway; OXPHOS, oxidative phosphorylation; TCA, tricarboxylic acid cycle; CYP, cytochrome P450; BCAA, branched-chain amino acid.

Supplementary Fig. S3

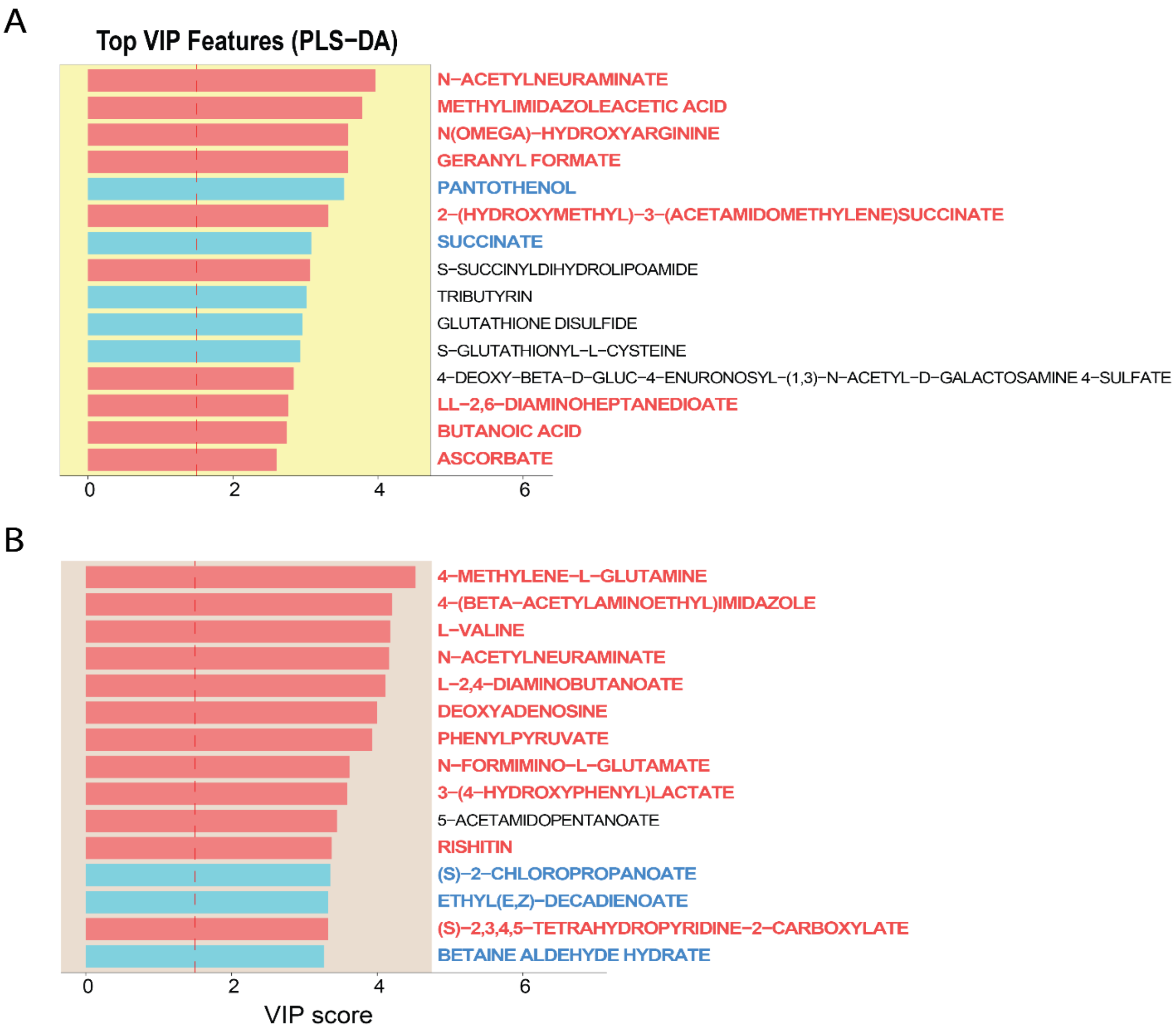

Supplementary Fig. S3 VIP-prioritized putatively annotated metabolite features in DRA KO colonic mucosa. Top VIP-ranked MSI level 3 metabolite features from the PLS-DA analysis of WT and DRA KO colonic mucosal metabolomic profiles. MSI level 3 features represent putatively annotated metabolites and were interpreted as exploratory feature-level support. Bar length indicates the VIP score, and the dashed vertical line indicates the VIP = 1.5 threshold used to prioritize discriminant features. Bar color indicates direction of change in DRA KO versus WT, with red indicating higher abundance in DRA KO and blue indicating lower abundance in DRA KO. Non-black metabolite labels indicate features that were also statistically significant after FDR correction (FDR < 0.05), with label color following the same direction as the bar. Black labels indicate VIP-prioritized features that did not reach FDR < 0.05. The prioritized putative features include signals consistent with glycan/mucin-associated metabolism, redox/glutathione metabolism, amino acid/nitrogen metabolism, nucleotide metabolism, and short-chain organic acid-related metabolism.

Supplementary Fig. S4

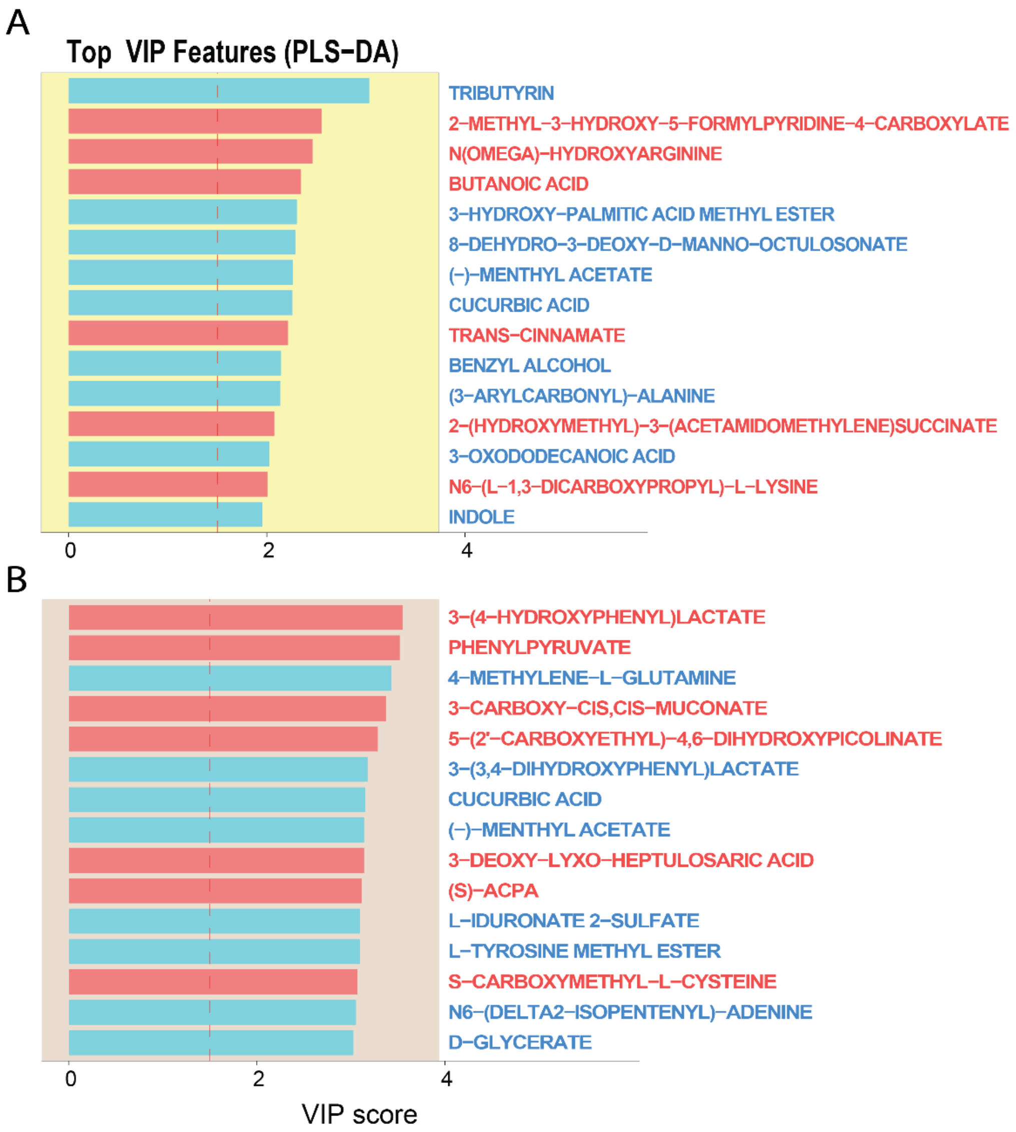

Supplementary Fig. S4 VIP-prioritized putatively annotated metabolite features in DRA KO feces. Top VIP-ranked MSI level 3 metabolite features from the PLS-DA analysis of WT and DRA KO fecal metabolomic profiles. MSI level 3 features represent putatively annotated metabolites and were interpreted as exploratory feature-level support. Bar length indicates the VIP score, and the dashed vertical line indicates the VIP = 1.5 threshold used to prioritize discriminant features. Bar color indicates direction of change in DRA KO versus WT, with red indicating higher abundance in DRA KO and blue indicating lower abundance in DRA KO. Non-black metabolite labels indicate features that were also statistically significant after FDR correction (FDR < 0.05), with label color following the same direction as the bar. Black labels indicate VIP-prioritized features that did not reach FDR < 0.05. The prioritized putative features include signals consistent with amino acid/nitrogen metabolism, aromatic amino acid-derived host-microbial metabolites, short-chain and organic acid metabolism, and lipid-associated metabolism.

Supplementary Fig. S5

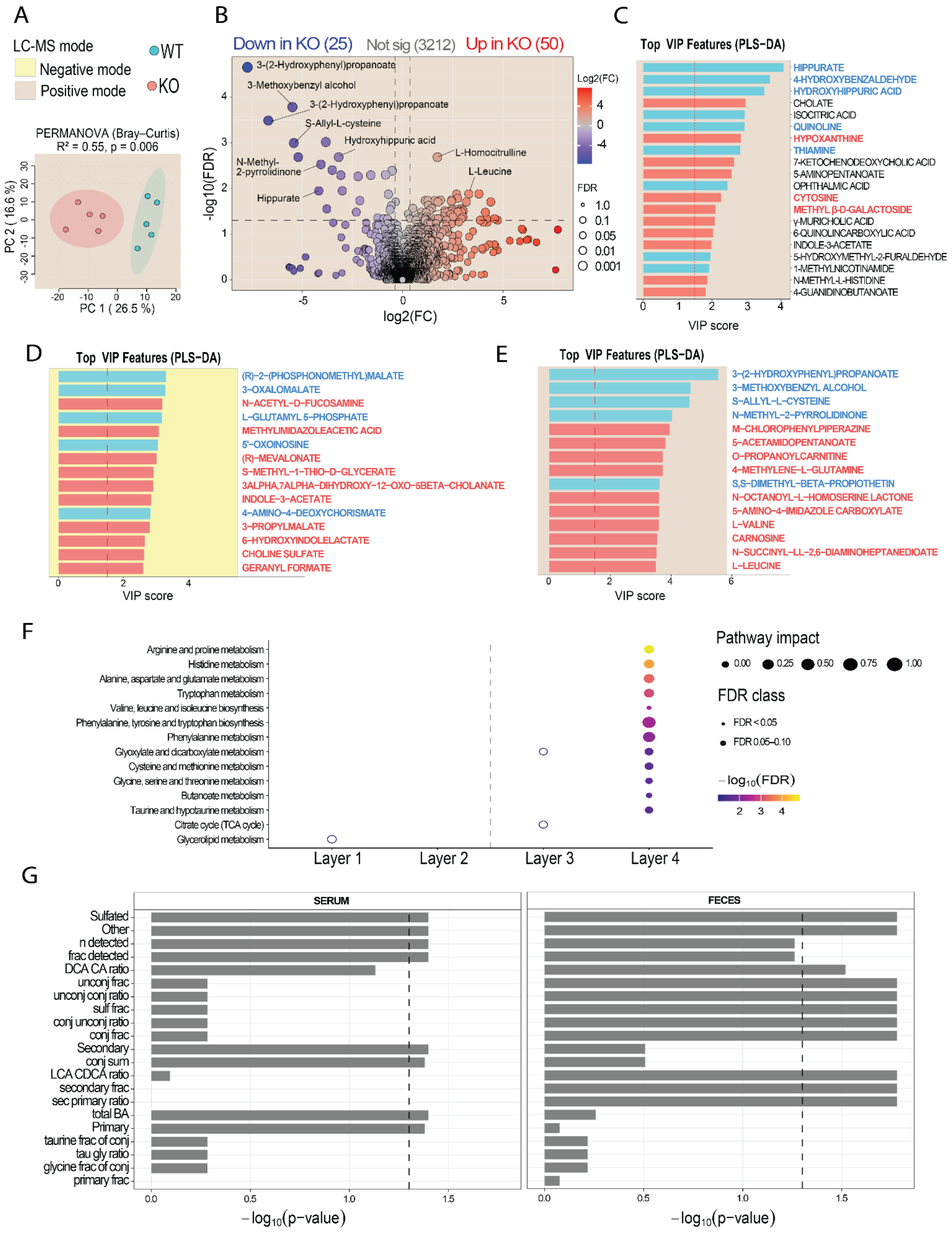

Supplementary Fig. S5 Additional serum metabolomic analyses and expanded bile acid metric summaries.

(a) Principal component analysis of serum metabolites acquired in positive-ion mode. Each point represents one biological replicate. WT and DRA KO samples are colored as indicated, and PERMANOVA statistics are shown. **(b)** Volcano plot of differentially abundant serum metabolite features in positive-ion mode. The x-axis shows $\log_2$ fold change in DRA KO versus WT, and the y-axis shows $-\log_{10}$ FDR. Numbers of significantly decreased and increased features in DRA KO are indicated, and representative metabolite features are labeled. **(c)** Top variable importance in projection (VIP)-ranked MSI level 1 serum metabolites identified by partial least-squares discriminant analysis (PLS-DA) in positive-ion mode. The dashed line indicates the VIP = 1.5 threshold used for prioritization. Bar color indicates direction of change in DRA KO versus WT, and non-black metabolite labels indicate features that also reached FDR significance, with label color following the same direction as the bar. **(d,e)** Top VIP-ranked MSI level 3 serum metabolite features from PLS-DA in negative-ion mode **(d)** and positive-ion mode **(e)**. MSI level 3 features represent putatively annotated metabolites and were interpreted as exploratory feature-level support. Bar length indicates VIP score, and the dashed line indicates VIP = 1.5. Bar color indicates direction of change in DRA KO versus WT, and non-black metabolite labels indicate features that also reached FDR significance. **(f)** Serum pathway topology analysis of altered serum metabolites across four complementary input layers: **Layer 1**, all significant annotated metabolites; **Layer 2**, significant MSI level 1 metabolites; **Layer 3**, VIP-prioritized MSI level 1 metabolites; and **Layer 4**, VIP-prioritized MSI level 3 metabolites. Dot size indicates pathway impact, dot color indicates $-\log_{10}$ FDR, and FDR class indicates pathways reaching FDR < 0.05 or suggestive FDR 0.05-0.10. **(g)** Class-level bile acid metrics altered in DRA KO mice in serum and feces. Bars show statistical significance as $-\log_{10}$(p-value). The dashed vertical line marks the nominal significance threshold of $p = 0.05$, and red labels denote metrics significantly altered in DRA KO relative to WT. **Abbreviations:** WT, wild-type; KO, knockout; DRA, down-regulated in adenoma; LC-MS, liquid chromatography-mass spectrometry; PERMANOVA, permutational multivariate analysis of variance; FDR, false discovery rate; FC, fold change; VIP, variable importance in projection; MSI, Metabolomics Standards Initiative; PLS-DA, partial least-squares discriminant analysis; BA, bile acid; DCA, deoxycholic acid; CA, cholic acid; LCA, lithocholic acid; CDCA, chenodeoxycholic acid; conj, conjugated; unconj, unconjugated; frac, fraction; sec, secondary; tau, taurine; gly, glycine; sum, total abundance/sum; n, number; sulf, sulfated.

Supplementary Fig. S6

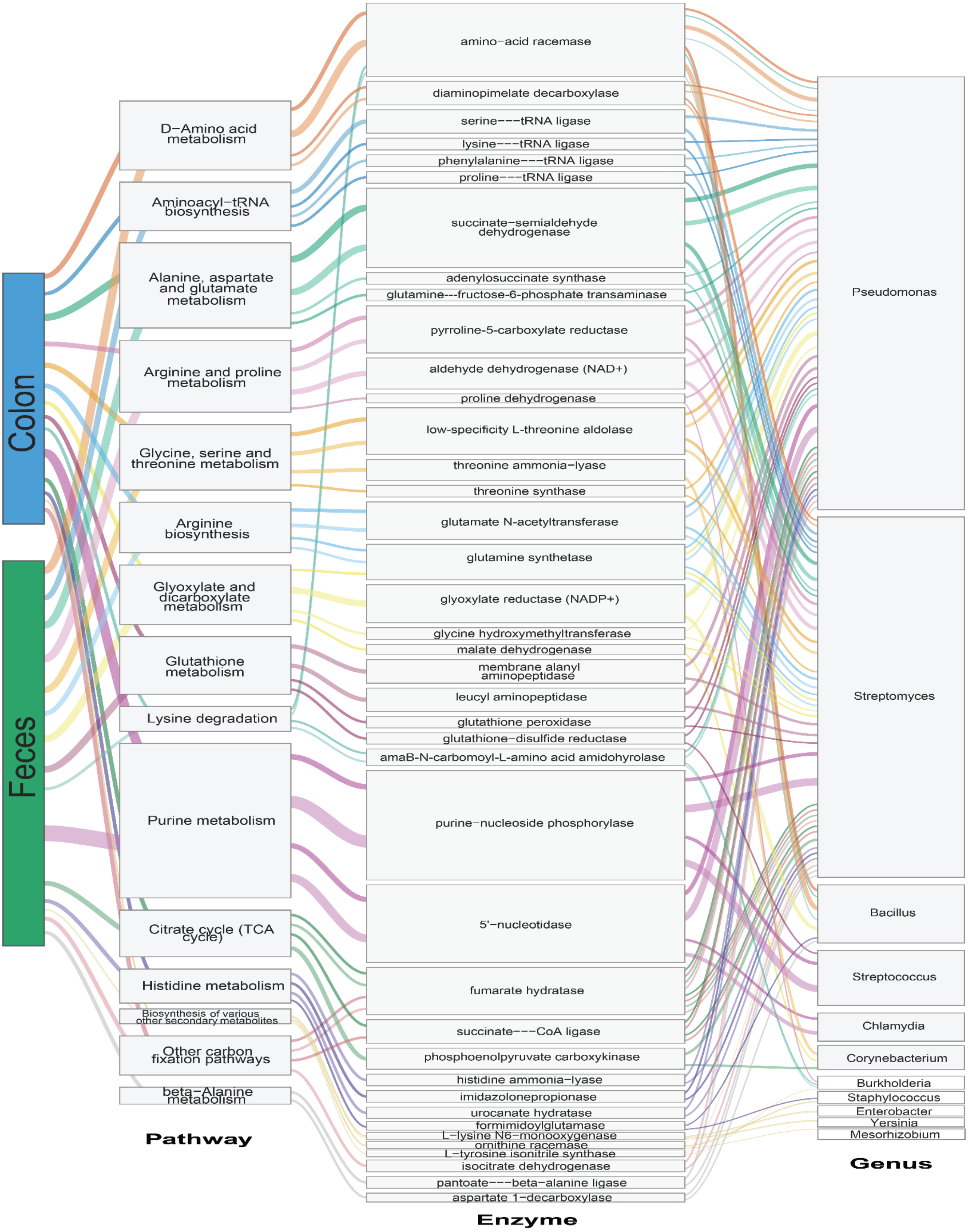

Supplementary Fig. S6 Expanded alluvial plot of the top 15 host-microbiota co-metabolism pathways in colon and feces linked to microbial enzymes and associated genera. Alluvial plot showing the top 15 inferred host-microbiota co-metabolism pathways identified in colon and feces and their connections to implicated microbial enzymes and associated microbial genera. Ribbon width reflects the relative contribution of each pathway-enzyme-genus connection.
